# SIGIRR deficiency contributes to CD4 T cell abnormalities by facilitating the IL1/C/EBPβ/TNF-α signaling axis in rheumatoid arthritis

**DOI:** 10.1186/s10020-022-00563-9

**Published:** 2022-11-18

**Authors:** Xiu Teng, Da-Chao Mou, Hui-Fang Li, Ling Jiao, Sha-Sha Wu, Jin-Kui Pi, Yan Wang, Meng-Li Zhu, Meng Tang, Yi Liu

**Affiliations:** 1 Laboratory of Human Disease and Immunotherapies, West China Hospital, Sichuan University, Chengdu, 610041 China; 2 Institute of Immunology and Inflammation, Frontiers Science Center for Disease-Related Molecular Network, West China Hospital, Sichuan University, Chengdu, 610041 China; 3 Core Facilities of West China Hospital, Sichuan University, Chengdu, 610041 China; 4 Rare Diseases Center, West China Hospital, Sichuan University, Chengdu, 610041 China

**Keywords:** Single immunoglobulin interleukin-1 receptor (SIGIRR), Rheumatoid arthritis (RA), CD4 T cells, Tumor necrosis factor (TNF), CCAAT/enhancer-binding protein β (C/EBP β)

## Abstract

**Background:**

Rheumatoid arthritis (RA) is a complex autoimmune disease with multiple etiological factors, among which aberrant memory CD4 T cells activation plays a key role in the initiation and perpetuation of the disease. SIGIRR (single immunoglobulin IL-1R-related receptor), a member of the IL-1 receptor (ILR) family, acts as a negative regulator of ILR and Toll-like receptor (TLR) downstream signaling pathways and inflammation. The aim of this study was to investigate the potential roles of SIGIRR on memory CD4 T cells in RA and the underlying cellular and molecular mechanisms.

**Methods:**

Single-cell transcriptomics and bulk RNA sequencing data were integrated to predict *SIGIRR* gene distribution on different immune cell types of human PBMCs. Flow cytometry was employed to determine the differential expression of SIGIRR on memory CD4 T cells between the healthy and RA cohorts. A Spearman correlation study was used to determine the relationship between the percentage of SIGIRR^+^ memory CD4 T cells and RA disease activity. An AIA mouse model (antigen-induced arthritis) and CD4 T cells transfer experiments were performed to investigate the effect of SIGIRR deficiency on the development of arthritis in vivo. Overexpression of *SIGIRR* in memory CD4 T cells derived from human PBMCs or mouse spleens was utilized to confirm the roles of SIGIRR in the intracellular cytokine production of memory CD4 T cells. Immunoblots and RNA interference were employed to understand the molecular mechanism by which SIGIRR regulates TNF-α production in CD4 T cells.

**Results:**

*SIGIRR* was preferentially distributed by human memory CD4 T cells, as revealed by single-cell RNA sequencing. SIGIRR expression was substantially reduced in RA patient-derived memory CD4 T cells, which was inversely associated with RA disease activity and related to enhanced TNF-α production. *SIGIRR*-deficient mice were more susceptible to antigen-induced arthritis (AIA), which was attributed to unleashed TNF-α production in memory CD4 T cells, confirmed by decreased TNF-α production resulting from ectopic expression of *SIGIRR*. Mechanistically, SIGIRR regulates the IL-1/C/EBPβ/TNF-α signaling axis, as established by experimental evidence and cis-acting factor bioinformatics analysis.

**Conclusion:**

Taken together, SIGIRR deficiency in memory CD4 T cells in RA raises the possibility that receptor induction can target key abnormalities in T cells and represents a potentially novel strategy for immunomodulatory therapy.

**Supplementary Information:**

The online version contains supplementary material available at 10.1186/s10020-022-00563-9.

## Introduction

Rheumatoid arthritis (RA) is a systemic autoimmune disease characterized by inflammatory synovitis and progressive joint destruction and is associated with irreversible disability and premature mortality (Smolen et al. 2018; Aletaha and Smolen 2018; Iain et al. 2011). During clinically apparent RA, innate and adaptive immune cells invade the synovium and together with joint-resident cells form tissue-destructive lesions, among which T cells are key pathogenic drivers required for autoantibody production and sustain synoviocyte proliferation, neoangiogenesis, and cartilage and bone erosions (Weyand and Goronzy 2021; Taylor et al. 2017; Firestein and McInnes 2017). Activated memory CD4 T cells migrating between the blood and synovium express cytokines (TNF-α, IFN-γ, IL-17, GM-CSF) and activation markers (receptor activator of nuclear factor κB ligand, osteoclast differentiation factor), contributing to tissue damage in RA (Wu et al. 2020; Emery et al. 2014; McInnes and Schett 2007; Jiang et al. 2020). Therefore, elucidating the abnormal profile of RA CD4 T cells under conditions of autoimmunity will lead to a better understanding of the mechanism by which the systemic immune response causes local joint disorders and will help to uncover potential drug targets against T cells for therapeutic strategies for RA.

SIGIRR (single immunoglobulin IL-1R-related receptor, also known as IL-1R8 or TIR8) is a member of the IL-1R superfamily (Garlanda et al. 2009; Molgora et al. 2016, 2018). Unlike classic IL-1R family members, SIGIRR consists of only a single extracellular Ig-like domain, a long cytoplasmic tail, and an intracellular Toll/IL-1R (TIR) domain (Guven-Maiorov et al. 2015). Several lines of evidence suggest that SIGIRR interferes with the association of TIR module-containing adaptor molecules with signaling receptor complexes of the ILR or TLR family, tuning downstream signaling, thus negatively controlling inflammatory and immune responses and T helper cell polarization and functions (Qin et al. 2005; Gulen et al. 2010). Moreover, IL-1R8 is the coreceptor of IL-18Rα for IL-37 and is required for the anti-inflammatory activity of this human cytokine (Nold-Petry et al. 2015; Lunding et al. 2015; Sánchez-Fernández et al. 2021; Li et al. 2021; Wang et al. 2022). Deregulated activation by TIR ligands in IL-1R8-deficient mice has been associated with exacerbated inflammation and immunopathology, including autoimmune diseases or selected cancers (Molgora et al. 2017; Riva et al. 2019; Xiao et al. 2007; Aiello et al. 2020; Wald et al. 2003; Giannoudaki et al. 2021). IL-1R8 is widely expressed, and its expression tends to decrease during the inflammatory response upon TIR ligation (Wald et al. 2003; Ueno-Shuto et al. 2014). Drexler et al. previously reported that the majority of SIGIRR seems to be expressed intracellularly in primary human monocytes and regulates inflammation in models of RA (Drexler et al. 2010). However, the roles of SIGIRR in the activation and cytokine production of CD4 T cells in RA have not been extensively investigated.

The current study identified loss-of-function of SIGIRR in CD4 T cells in patients with RA and a murine model of antigen-induced arthritis. Mechanistically, we discovered an unconventional SIGIRR/C/EBPβ signaling axis that directly impacts TNF-α production in CD4 T cells, contributing to articular inflammation. These results demonstrate a role for SIGIRR as a negative modulator of RA inflammation, probably through its modulation of IL-1 signaling in CD4 T cells.

## Materials and methods

### Single cell RNA-Seq analysis

A single-cell transcriptomics dataset for peripheral blood mononuclear cells (PBMCs) derived from four healthy human donors was obtained from the Gene Expression Omnibus (GSM3087619, GSM3087622, GSM3087624, GSM3087626). Data preprocessing, including cell quality control, has been described in a previous study (Chen et al. 2018). Principal component analysis was performed on quality cells, followed by unsupervised hierarchical clustering by t-distributed stochastic neighbor embedding algorithm (t-SNE) analysis. The marker genes used for cluster annotation are listed in Additional file 8: Table S1 (Zheng et al. 2017; Li et al. 2019; Sinha et al. 2018; Karamitros et al. 2017). The top genes displayed in the heatmap have the most significant *P* value and are differentially expressed marker genes for each cell type. Violin plots showing the expression levels of marker genes or SIGIRR by different populations of PBMCs.

### Bulk transcript profiling (RNA-Seq) for immune lineages in blood and PBMCs in human

The bulk RNA-seq datasets for major immune lineages in whole blood and PBMCs are available from v21.0.proteinatlas.org (Uhlen et al. 2019). The read counts were normalized to transcripts per million protein coding genes (pTPM) for each cell type. The gating strategy for flow sorting is indicated in Additional file 9: Table S2.

### Healthy donor and patient samples

Cryopreserved peripheral blood for flow cytometry analysis and sorting was collected at the Department of Rheumatology, West China Hospital, Sichuan University. This study was approved by the West China Hospital Ethical Vetting Board, Sichuan University (number 2018–199). Diagnosis for RA was based on ACR (American College of Rheumatology)/EULAR (European League Against Rheumatism) 2010 rheumatoid arthritis classification criteria (Kay and Upchurch 2012). The demographic and clinical laboratory data of these patients were obtained from hospital records and are summarized in Additional file 10: Table S3. RA cases and controls did not reveal any significant differences with regard to age and sex. DAS28 (CRP) = 0.56*√(TJC28) + 0.28*√(SJC28) + 0.014*GH + 0.36*ln(CRP + 1) + 0.96, where TJC = tender joint count and SJC = swollen joint count (Wells et al. 2009).

### Cell preparation

Total peripheral blood mononuclear cells (PBMCs) were isolated from fresh blood of healthy volunteers and RA patients by density gradient centrifugation using Percoll (Solarbio). Human PBMCs were kept in liquid nitrogen before thawing in a 37 °C water bath. Cells were resuspended in T-cell culture medium (RPMI-1640, 10% fetal bovine serum (HyClone), 2 mM L-glutamine (Solarbio), 100 U/ml penicillin, 100 μg/ml streptomycin (both Solarbio), and 5 mM 2-mercaptoethanol (Sigma–Aldrich)). Samples were then rested overnight at 37 °C before restimulation with 50 ng ml ^−1^ phorbol 12-myristate 13-acetate (PMA, Sigma–Aldrich) and 500 ng ml^−1^ ionomycin (ION, Sigma–Aldrich) in the presence of brefeldin A (BD Biosciences) for 4 h at 37 °C. Synovial tissue samples were disaggregated into single-cell suspensions as previously described (Carter et al. 2011). Murine synovial tissues were isolated from the knee joints of AIA mice and incubated with 1 mg/mL collagenase IV (Thermo Fisher Scientific) and 100 μg/ml DNase I (Roche) in RPMI in a 37 °C water bath for 45 min. Single-cell suspensions were obtained by passing cells through 70 μm cell strainers for flow cytometry staining.

### Flow cytometry

For surface staining, the single-cell suspensions were washed and stained with fluorescence-conjugated antibodies for 15 min at room temperature. For intracellular cytokine staining, the combined surface- and live/dead-stained sample was fixed with fixation buffer (Thermo Fisher Scientific) for 20 min at room temperature. Fixed cells were washed twice with permeabilization buffer (Thermo Fisher Scientific). Cells were resuspended in 100 μl of intracellular antibody mixture in permeabilization buffer for 30 min at room temperature. The sample was washed, the supernatant was removed, and the cells were resuspended in cell staining medium. All fluorescence-conjugated antibodies used for flow cytometry are listed in Additional file 11: Table S4. Cells were acquired and analyzed with FACSAria SORP flow cytometers (BD Biosciences). All flow cytometry data were analyzed with NovoExpress software (Agilent). Memory CD4 T cells (CD4^+^ CD45RO^+^ CD45RA^−^) from both healthy and RA cohorts were separated by flow-assisted cell sorting (FACSAria SORP, BD Biosciences) for subsequent stimulation in cell culture.

### T cell culture ex vivo

Sorted human memory CD4 T cells were cultured in 48-well plates (5 × 10^5^ cells per well) containing plate-bound anti-CD3 (5 μg/ml, BD Bioscience) and soluble anti-CD28 (1 μg/ml, BD Bioscience) supplemented with 20 ng/ml IL-1β (Peprotech). For quantitative real-time PCR and ELISA, cells or supernatants were collected on days 1 and 2. Murine splenic CD4 T cells were enriched by a mojoSort™ mouse CD4 T cell isolation kit (Biolegend), cultured with plate-bound anti-CD3 (2 μg/ml, BD Bioscience) and soluble anti-CD28 (1 μg/ml, BD Bioscience), and stimulated with 20 ng/ml IL-1β (Peprotech) for 30 min to study the phosphorylation of signaling molecules or 48 h for cytokine production by ELISA or flow cytometry.

### Mouse primary CD4 T cell transfection

Mouse primary CD4 T cells were transfected with Dharmacon Accell nontargeting control (Horizon Discovery, D-00190–10) or mouse C/EBPβ-specific siRNA pool (composed of the following four antisense sequences: I, UGGUUUACAUGUCGACUAA; II, UGGUUUACAUGUUUUCUGA; III, UGGUUUACAUGUUUUCCUA; IV, UGGUUUACAUGUUGUGUGA. Horizon Discovery, E-043110–00) using Accell delivery medium, following the manufacturer’s instructions.

### Activation of memory CD4 T cells ex vivo

Memory CD4^+^ T cells were flow-sorted from splenocytes of CFA/mBSA-immunized mice as responder cells. To obtain stimulatory cells (providing additional costimulatory signals for T cell activation), single splenocyte suspensions were obtained from synergetic mice and treated with red blood cell lysis solution at room temperature for 5 min to remove erythrocytes, followed by mitomycin C treatment at 37 ℃ for 20 min, washed and used as stimulatory cells. Then, responder cells (1 × 10^5^) were cultured with mitomycin C-treated stimulatory cells (5 × 10^5^) in 96-well flat-bottom plates in the presence of 25 μg/ml soluble mBSA at 37 ℃ for 3 days. The procedures to establish the coculture system of stimulatory cells/responder cells were previously described (Kruisbeek et al. 2004; Wong et al. 2009) with minor modifications. Cytokine production in memory CD4 T cells in this coculture system was analyzed with intracellular staining by flow cytometry.

### ELISA

Cytokine production of human or mouse CD4 T cell culture supernatants was measured using uncoated ELISA kits (Thermo Fisher Scientific) or specific mouse ELISA kits (Neobioscience). All procedures were performed as recommended by the manufacturer.

### Plasmids, retrovirus production and transduction

The SIGIRR cDNA was PCR amplified and cloned into pMIG (SIGIRR-IRES-GFP). Phoenix helper-free retrovirus producer lines (Ivanov et al. 2006; Schraml et al. 2009) were transfected with 10 μg of the indicated plasmids using polyethylenimine (Polysciences). The viral supernatant was collected and supplemented with 5 mg/ml polybrene (Yeasen). For viral transduction, sorted mouse CD4 T cells were plated, fresh retrovirus supernatant was added, and the cells were spun at 2500 rpm for 1.5 h at 30 °C. After spin infection, the cells were cultured in T cell culture medium and harvested on day 3 for intracellular cytokine staining and quantitative real-time PCR analysis.

### Generation of *Sigirr*^−/−^ mice

*Sigirr* ^+*/−*^ founder mice on a C57BL/6 background were generated and purchased from Cyagen Biosciences. Male founder mice were bred with female mice to generate *Sigirr* ^+*/*+^ and *Sigirr *^*−/−*^ mice. SIGIRR KO mice were identified by genotyping PCRs with F1, F2 and R1 primers (listed in Additional file 12: Table S5) performed on tails at 3–4 weeks of age. Mice were housed in an animal facility (SPF condition) at Sichuan University in compliance with the guidelines set by West China Hospital Institutional Animal Care and Use Committee, Sichuan University.

### Induction of antigen-induced arthritis (AIA)

AIA was induced as previously described (Engdahl et al. 2013). C57/BL6 mice were immunized with 0.2 mg of methylated bovine serum albumin (mBSA; Sigma–Aldrich) dissolved in phosphate buffered saline (PBS) and emulsified with an equal volume of Freund’s complete adjuvant (Sigma–Aldrich). One week later, the mice received a second injection of 0.3 mg of mBSA dissolved in 30 μl of vehicle into the right knee joint (day 0). The left knee was injected with vehicle and used as an internal control. Animals were inspected daily for arthritis development by measuring the knee joint diameter using a micrometer. Arthritis severity was assessed through a comparison between the experimental right limb and the control left limb.

### Histology and immunohistochemistry of inflammatory arthritis

Four days after the intra-articular injection of mBSA, the mice were anesthetized and sacrificed. Knees from the immunized mice were separately placed in 4% formaldehyde, decalcified, and embedded in paraffin. Sections were stained with hematoxylin and eosin. For immunohistochemical staining, 5-μm-thick sections of paraformaldehyde-fixed mouse joints were deparaffinized and hydrated with distilled water. Heat-induced epitope retrieval was performed by incubation in citrate buffer (pH = 6.0) at 98 °C for 5 min, and endogenous peroxidase was quenched by treatment with 3% H_2_O_2_ in PBS for 5 min. Next, goat serum (ZSGB-Bio) was applied for 30 min to block nonspecific binding on the tissue sections. CD4 T cells in the synovium were detected by incubation with a rabbit anti-CD4 primary antibody (Abcam) overnight at 4 °C in a humidified chamber. After washing with PBS, the histological slides were incubated with biotinylated anti-rabbit secondary Ab (ZSGB-Bio) and HRP-labeled streptavidin complex (ZSGB-Bio) for 30 min. The staining reaction for CD4 was visualized by a DAB Peroxidase Substrate Kit (MXB Biotechnologies), and positive signals of DAB chromogen were developed as brown precipitates. The slides were counterstained with hematoxylin (Beyotime Biotechnology) to detect nuclei. Images of the stained slides of joints were obtained with a ZEISS microscope. Five random fields of view (400 ×) of each mouse psoriatic plaque section stained with H&E were assessed using the Baker Score system. Similarly, mast cells were quantified by counting five randomly selected fields in brown-stained slides. Immunohistochemical staining was quantified by computer-assisted methods.

### Isolation of total and Naïve CD4^+^ T Cells

CD4^+^ T cells were purified from spleens using anti-CD4 magnetic microbeads (Biolegend) (purity > 90%). For naïve CD4^+^ T cells, single-cell suspensions were first incubated with Fixable Viability Stain 780 (BD Bioscience, 1:1000) to discriminate live cells, followed by incubation with anti-CD25-APC, anti-CD4-BV421, anti-CD62L-PE, and anti-CD44-PerCP/Cy 5.5. Cell sorting was performed on an ARIA II SORP cytometer (BD Bioscience) to obtain a pure population of naïve CD4^+^ CD25^−^ CD44^−^CD62L^+^ T cells (> 97% purity). A total of 5 × 10^6^ naïve CD4 T cells derived from the spleen of *Sigirr* ^+/+^ or *Sigirr *^−/−^ mice were intravenously transferred into *Rag1*^*−/−*^ mice. One day later, the recipient mice were immunized with mBSA as described above.

### Quantitative real-time PCR (QRT-PCR)

Total RNA was extracted from tissues using TRIzol (Thermo Fisher Scientific), reverse-transcribed into cDNA using PrimeScript™ RT reagent Kit with gDNA Eraser (Takara) and followed by PCR amplification in triplicate using ChamQ SYBR qPCR Master Mix (Vazyme) in the CFX96 touch real-time PCR detection system (Bio-Rad). The primer sets for real-time PCR can be found in Additional file 12: Table S5.

### Immunoblots

Murine CD4 T cells were collected after 30 min of treatment with IL-1, and total cell lysates were subjected to SDS–PAGE and then blotted using the indicated antibodies (Additional file 13: Table S6). The quantitative analysis of integral OD of the bands in the Western blot was performed using.

ImageJ software.

### Transcriptional regulation of C/EBPβ on TNF gene

DNA binding motifs of transcription factor C/EBPβ were predicted by retrieving JASPAR, the online collection of transcription factor DNA-binding preferences (Fornes et al. 2020). Experimental binding of C/EBPβ to *TNF* promotor regions was revealed by ChIP-seq data deposited in the Cistrome Data Browser (Zheng et al. 2020).

### Statistical analysis

All statistical analyses were performed with GraphPad Prism software. Analysis of the differences in SIGIRR expression and intracellular cytokines between healthy and RA populations was assessed using the Mann–Whitney test, and the relationship between SIGIRR expression and disease activity DAS28 was established by Spearman’s correlation test. Other comparison analyses in this study were assessed by unpaired t test (between two groups) or by multiple t tests using the Holm‒Sidak Method as indicated.

## Results

### SIGIRR is an essential regulator of memory CD4 T cells

**Fig. 1:**
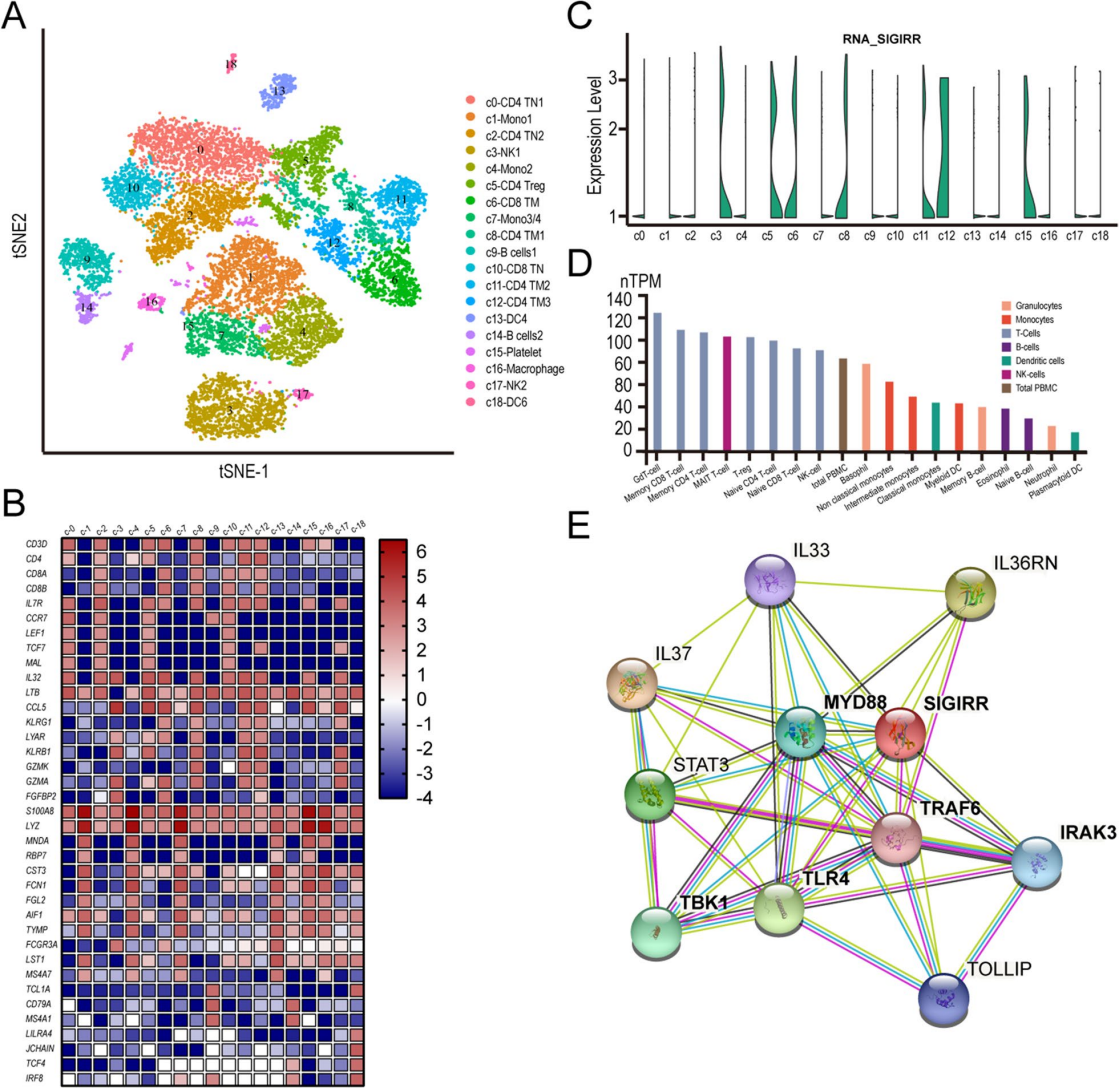
SIGIRR expression in human PBMCs and its protein interaction. *SIGIRR* expression in healthy human PBMCs revealed by single-cell RNA-seq (**A**–**C**, GSE112845) and bulk RNA-seq (**D**). **A** 2D visualization of single-cell clusters in healthy human PBMCs by t-SNE. Each dot represents a single cell. **B** Heatmap of 19 cell clusters identified by well-known unique signature genes. The Ζ-score, calculated on the basis of the permutation *P* value, is listed on the right side to show the gene expression level. **C** Violin plots showing the expression profile of *SIGIRR* across 19 clusters in human PBMCs. **D** Normalized transcript expression values (pTPM, transcripts per kilobase million) of *SIGIRR* in 18 hematopoietic cell types and total PBMCs of healthy donors by bulk RNA sequencing (available from v21.0.proteinatlas.org.). **E** SIGIRR network interaction revealed by the STRING online database

To reveal the intrinsic structure and potential functional subtypes of the overall T cell populations, the center of the immune response, we first explored *SIGIRR* expression in human peripheral blood mononuclear cells (PBMCs) using a scRNA-seq atlas of human PBMCs generated by Chen et al*.* (Chen et al. 2018). We performed unsupervised clustering of PBMCs using the t-SNE algorithm (Fig. 1A). A total of 19 stable clusters (c0-c18) emerged, including 6 clusters for CD4 T cells, 2 clusters for CD8 T cells, 3 clusters for monocytes, 2 clusters for dendritic cells, 1 cluster for macrophages and 1 cluster for platelets (unremoved parts when isolating PBMCs from whole blood), each with its unique signature genes, as shown in Fig. 1B, Additional file 1: Figure S1 and Additional file 8: Table S1.

Cells of the c0 and c2 CD4 clusters specifically expressed ‘‘naïve’’ marker genes such as *TCF7* and *LEF1* (Additional file 1: Figure S1 and Additional file 8: Table S1). Cluster 5 was characterized by high expression of *IL32, LTB* and *IL7R*, which are commonly associated with CD4 T cells with regulatory functions. Cluster 8 was characterized by specific expression of *KLRG1, CCL5, LYAR and IL7R*, thus representing memory CD4 T cells. The remaining CD4 cells, falling into clusters c11 and c12, exhibited high expression of cytotoxic molecules, including *KLRB1, GZMK,* and *CCL5*, indicative of the status of cytotoxic CD4 T cells. However, no clusters for γδT cells and mucosal-associated invariant T cells were identified due to their small proportions of PBMCs. Similarly, we identified two major CD8 T cell clusters, c6 with naïve marker genes and c10 with cytotoxic marker genes, representing naïve and memory CD8 T cells, respectively. Gene markers used for defining B cells, natural killer cells, monocytes and dendritic cells were described in previously published studies (Zheng et al. 2017; Villani et al. 2017).

We notified the preferential expression of *SIGIRR* in NK cells (c3), memory CD4 (c8, c11 and c12) or CD8 T cells (c6, Fig. 1 C) rather than monocytes (c1, c4 and c7) or dendritic cells or macrophages (c13, c18 and c16), as shown by violin plots in Fig. 1C. Bulk RNA sequencing data for specific cell types validated the contribution of natural killer cells, memory CD4 T cells and memory CD8 T cells to *SIGIRR* mRNA production (Fig. 1 D). Thus, the expression atlas of SIGIRR in healthy human PBMCs prompted us to focus on the function of SIGIRR in memory CD4 T cells, which are critical for host defense but are also major drivers of immune-mediated diseases (O'Shea and Paul 2010; Borst et al. 2018; Beek et al. 2021), such as RA (Jiang et al. 2020; Raphael et al. 2020). STRING, a powerful online tool to provide an overview of protein–protein interactions, showed that SIGIRR was an essential regulator of the MyD88-dependent toll-interleukin receptor signaling pathway (Fig. 1 E), which was enriched in RA (Zhang et al. 2019). The pathogenicity of RA CD4 T cells is linked to tissue invasiveness combined with invasive membrane ruffles. Therefore, we focused on the possible differential expression of SIGIRR in memory CD4 T cells between healthy and RA cohorts in this study and the underlying immune regulation in RA.

### Downregulation of SIGIRR in memory RA CD4 T cells was related to enhanced production of TNF-α

**Fig. 2:**
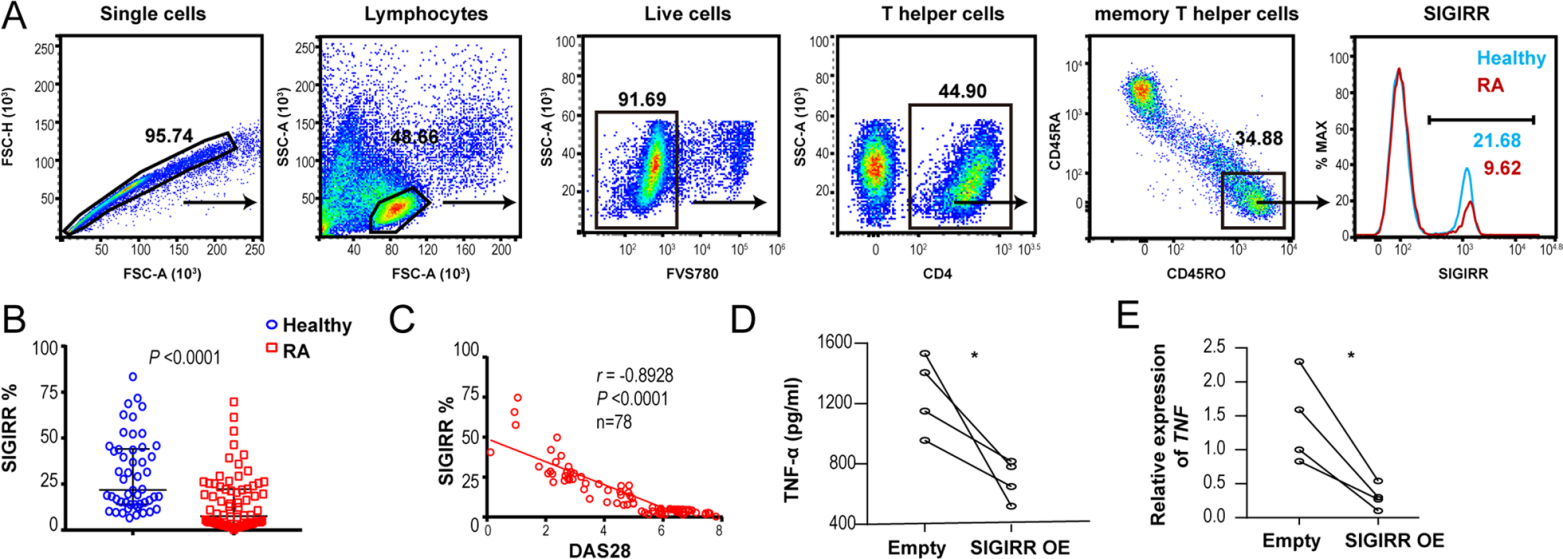
Frequencies of SIGIRR-positive memory CD4 T cells in PBMCs of patients with RA and the healthy population. Related to Additional file 1: Figure S2. **A**, **B** Gating strategies used to analyze SIGIRR expression in memory CD4 T cells in human peripheral blood mononuclear cells (PBMCs). Memory CD4 T cells were identified as CD4 ^+^ CD45RO ^+^ CD45RA ^−^ previously gated on single live (FVS780 ^−^) lymphocytes (FSC ^low^ SSC ^low^). Representative flow cytometry histogram plots showing SIGIRR expression by memory CD4 T cells in human PBMCs in healthy and RA cohorts (n = 50 for healthy and 78 for RA individuals), with quantification of results as frequency (**B**). **C** Linear regression analysis of the proportion of SIGIRR in memory CD4^+^ T cells and disease activity score 28 (DAS28) of 78 RA patients (*r* =  − 0.8928, *P* < 0.0001, n = 78). **D**, **E** SIGIRR^−^ memory CD4 T cells in RA PBMCs (n = 4) were flow-sorted and transduced with retroviral empty vectors (empty) or vectors encoding SIGIRR (SIGIRR OE). Then, SIGIRR^−^ memory CD4 T cells (1 × 10^6^ cells/mL) were treated with IL-1β in the presence of α-CD3/α-CD28 antibodies for 48 h. TNF-α production in the supernatants of the SIGIRR^−^ memory CD4^+^ T cell cultures stimulated with was measured by ELISA (**D**), and the relative expression of TNF-α was monitored by quantitative real-time RT‒PCR (**E**). Each symbol **B**, **C** represents an individual human; small horizontal lines indicate the median ± interquartile (**B**). *P < 0.05 and ****P < 0.0001, Mann‒Whitney test (**B**), Spearman’s correlation (**C**) or paired t test (**D**, **E**)

To determine the distribution of SIGIRR in memory CD4 T cells, we investigated the extracellular frequencies of SIGIRR in CD4 T cells from RA patients and healthy controls by flow cytometry and observed a significantly lower proportion of SIGIRR-positive memory CD4 T cells (CD4 ^+^ CD45RO ^+^ CD45RA ^−^) in RA cohorts (Fig. 2 A, B) (median = 7.6%) than in healthy controls (median = 21.9%). A correlation study showed a negative relationship between the frequency of SIGIRR-positive memory CD4 T cells and the DAS28 score (Fig. 2 C, *r* = -0.8828, *P* < 0.0001), indicating that decreased SIGIRR expression in memory CD4 T cells was negatively associated with active RA disease activity. Additionally, patients with seropositive anti-CCP antibody showed a lower frequency of SIGIRR expression in memory CD4 T cells in PBMCs (Additional file 2: Figure S2), implying the contribution of antigen-specific CD4 T cells to the reduced expression level of SIGIRR in RA. We next examined the effect of reduced SIGIRR expression in memory CD4 T cells on the production of essential cytokines in RA. PBMCs from RA patients showed markedly higher frequencies of SIGIRR^+^ memory CD4 T cells secreting TNF-α than those from healthy individuals (median 39.4% *vs* 18.6%, Additional file 3: Figure S3 A-B) and a slight increase in IL-17-producing cells (median 0.83% *vs* 0.63%, Additional file 3: Figure S3 A-B). Overall, significantly lower expression of TNF-α was observed on SIGIRR^+^ memory CD4 T cells than on SIGIRR^−^ memory CD4 T cells in both healthy individuals and RA patients. It was also interesting that TNF-α expression on SIGIRR^+^ memory CD4 T cells was much lower in RA patients than in healthy individuals (Additional file 3: Figure S3 C).

To clearly confirm the suppression of SIGIRR on TNF-α expression in memory CD4^+^ T cells ex vivo, RA SIGIRR^−^ memory CD4^+^ T cells from PBMCs were flow-sorted and transduced with retroviral empty vectors (empty) or vectors encoding SIGIRR (SIGIRR overexpression) and treated with IL-1β in the presence of α-CD3/α-CD28 antibodies for 48 h. As shown in Fig. 2 D and E, overexpression of SIGIRR in RA SIGIRR^−^ memory CD4^+^ T cells was associated with a significant decrease in TNF-α production. Thus, the decreased frequency of SIGIRR in memory CD4 T cells and its potential regulation of TNF-α in RA patients imply that SIGIRR could play a pivotal role in the pathogenesis of RA.

### Mice with SIGIRR deficiency are susceptible to antigen-induced arthritis

**Fig. 3:**
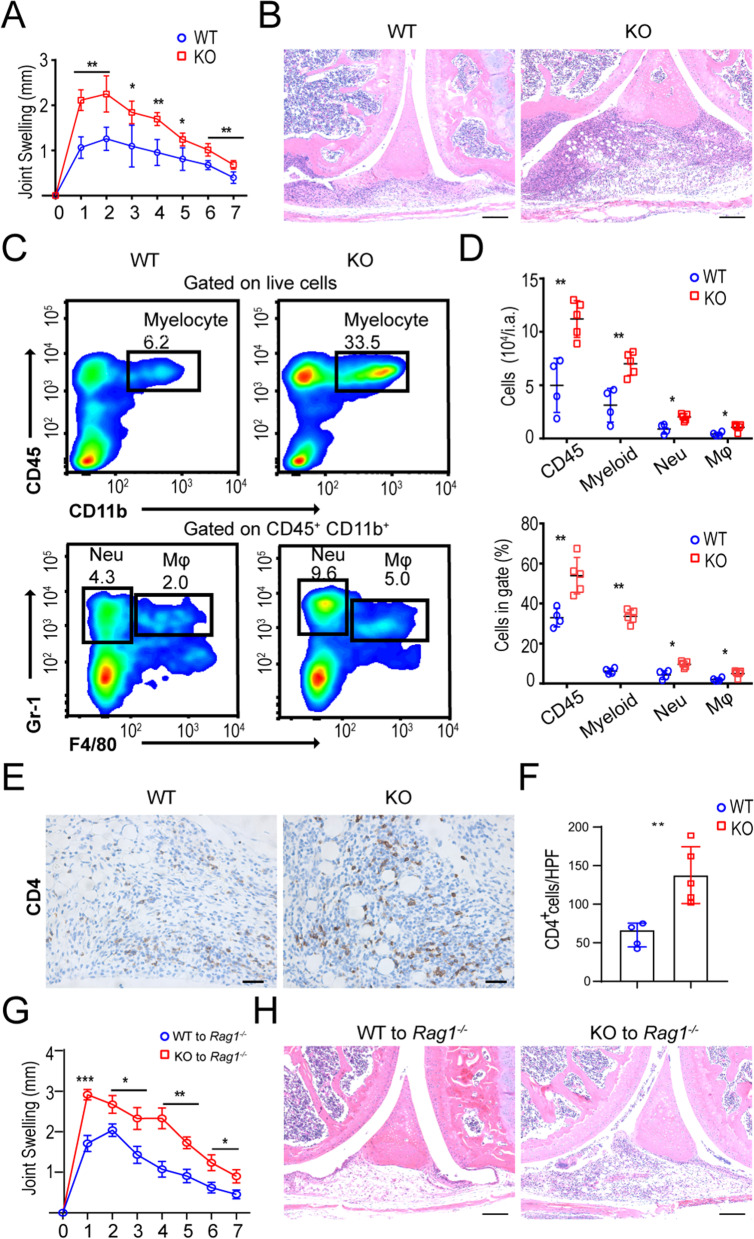
Development of AIA in wild-type and SIGIRR-deficient C57BL/6 mice. **A** Knee joint swelling curves of WT and SIGIRR KO mice (n = 4–5) during AIA. The development of arthritis was monitored daily by measuring knee joint swelling. **B** H&E staining of joints from WT and SIGIRR KO mice challenged with mBSA; scale bar, 100 μm. **C**, **D** Flow cytometry density plots of myeloid cells gated on live single cells and granulocyte or macrophage subsets gated on myeloid cells in synovial tissues with quantification of results as absolute number per inflammatory articulate (i.e., **D**) or frequency among gated cells (**D**). **E** Immunohistochemistry images of joint sections stained with CD4 T cells; scale bar, 20 μm. **F** CD4-positive cells quantitated by counting brown dots from four to five blind different sections per mouse as shown in **E**. **G** Knee joint swelling curves of *Rag1*^*−/−*^ mice transferred with CD4 T cells from WT (WT to *Rag1 *^−/−^) or SIGIRR KO (KO to *Rag1 *^−/−^) mice (n = 4) during AIA. **H** H&E staining of joints in *Rag1*^*−/−*^ mice adoptively transferred with CD4 T cells. Data are representative of two independent experiments, scale bar 100 μm

To assess the function of SIGIRR in the immune response, we generated SIGIRR-deficient mice (*Sigirr *^−/−^, Additional file 4: Figure S4A, B) as described in the Materials and Methods. The progeny of SIGIRR-deficient mice were healthy and showed no notable abnormalities. However, adult *Sigirr *^−/−^ mice displayed obvious splenomegaly (Additional file 4: Figure S4C) in a physiological state, consistent with the role of SIGIRR as a negative regulator in immunology. After analyzing the composition of splenic immune cells and CD4 T cell subpopulations, we observed a higher fraction of granulocytes (Additional file 5: Figure S5A-D) and enhanced maturation of CD4^+^ T cells (Additional file 5: Figure S5E-H) in *Sigirr *^*−/−*^ mice than in *Sigirr* ^+*/*+^ mice, consistent with the role of SIGIRR as a negative regulator in immunology. To examine the role of endogenous SIGIRR during inflammation in vivo, we investigated the clinical and histopathological features of SIGIRR-deficient C57BL/6 mice in a mouse model of antigen-induced arthritis (AIA) with mBSA as an antigen, which shares both immunological and pathological features with human rheumatoid arthritis. *Sigirr *^−/−^ mice developed more severe knee joint swelling (Fig. 3A). Histological analysis of whole ankle joints demonstrated a significant increase in the infiltration of polymorphonuclear leucocytes and synovial exudate compared with their *Sigirr* ^+*/*+^ littermates (Fig. 3B). To further characterize the cellular and molecular pathways affected by SIGIRR deficiency, we characterized and quantitated the inflammatory synovial infiltrate by flow cytometry. The synovium of *Sigirr *^−/−^ mice showed a remarkable infiltration of CD45^+^ leucocytes, especially inflammatory neutrophils, macrophages (Fig. 3C-D) and CD4 T cells (Fig. 3E, F), which was an almost twofold enhancement compared with that in WT mice. To test the intrinsic role of SIGIRR in CD4 T cells, we sorted naïve T cells (CD4^+^CD44^lo^) from WT and *Sigirr *^−/−^ mice and transferred them into *Rag1 *^−/−^ mice (Additional file 6: Figure S6). Upon immunization with mBSA, mice with *Sigirr *^−/−^ CD4 T cells developed more severe joint swelling than did the mice with *Sigirr* ^+*/*+^ CD4 T cells (Fig. 3G). Consistent with the clinical symptoms, the infiltration of leucocytes was significantly heightened in the joints of *Rag1 *^−/−^ recipient mice adoptively transferred with *Sigirr *^−/−^ CD4 T cells compared to mice adoptively transferred with *Sigirr* ^+*/*+^ CD4 T cells (Fig. 3H). Collectively, these data obtained in vivo suggest that the loss of SIGIRR expression in CD4 T cells results in enhanced susceptibility to antigen-induced arthritis.

### SIGIRR suppressed the production of TNF-α in mBSA-specific CD4 T cells

**Fig. 4:**
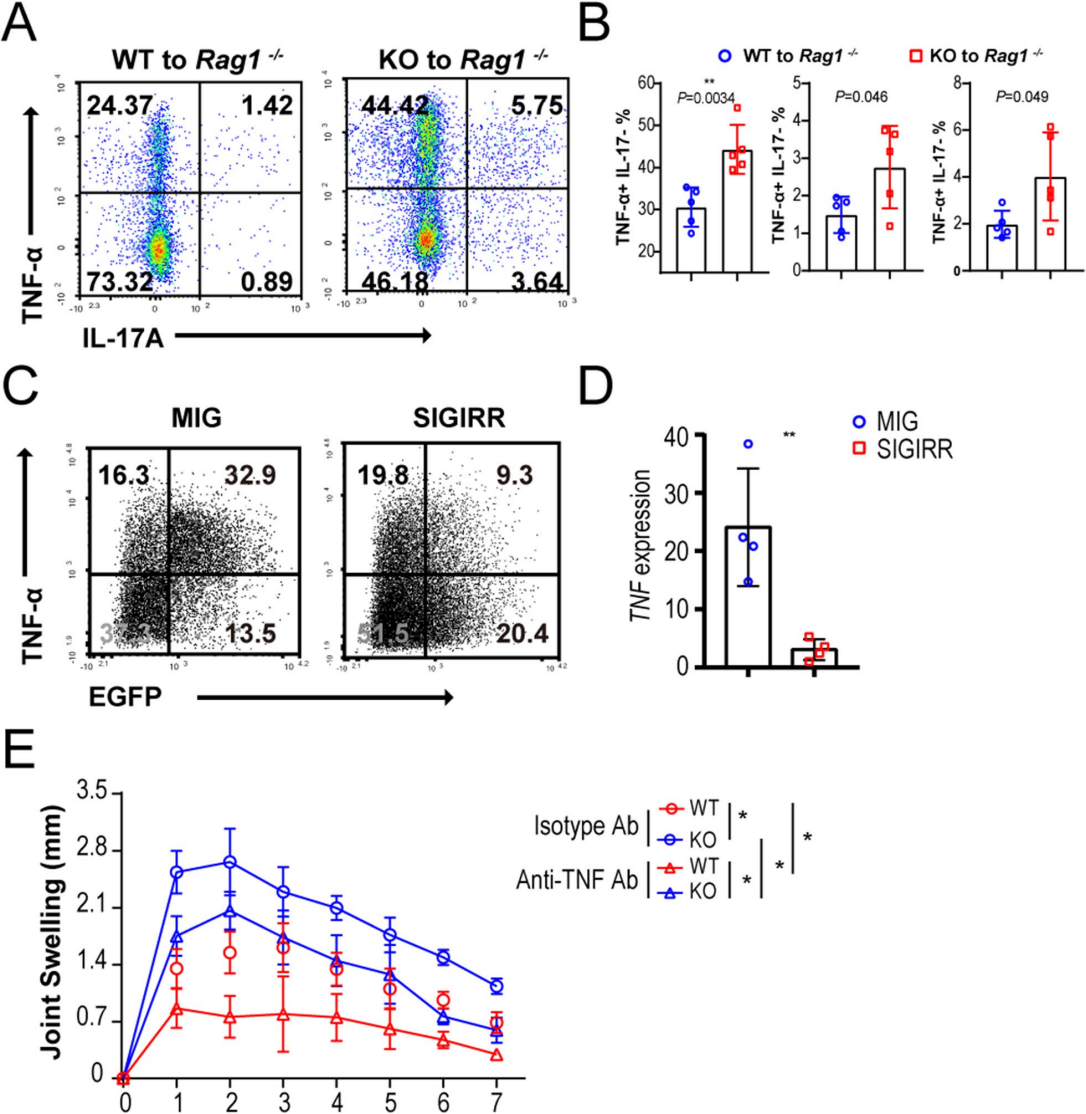
Production of TNF-α in mBSA-specific CD4 T cells regulated by deficiency or ectopic expression of SIGIRR. **A**, **B** Intracellular production of TNF-α and IL-17 among memory CD4 T cells (CD4^+^ CD44^hi^) sorted from immunized *Rag1*^*−/−*^ mice transferred with SIGIRR (WT to *Rag1 *^−/−^) or SIGIRR KO (KO to *Rag1 *^−/−^) CD4 T cells and then cultured by stimulation with mBSA in the presence of mitomycin C-treated syngeneic splenocytes for 3 days. **A** Numbers in quadrants indicate percent TNF-α^+^ IL-17A^−^ cells (top left) or TNF-α^−^ IL-17A^+^ cells (bottom right) or TNF-α^+^ IL-17A^+^ (top right) gated in CD4^+^ CD44^hi^ cells. **B** Each symbol represents an individual mouse; small horizontal lines indicate the mean ± SD. **P < 0.01, *P < 0.05 (*n* = 5, unpaired t test); Data are from one experiment representative of two independent experiments with similar results. **C** Intracellular TNF-α production in sorted memory CD4 T cells isolated from wild-type C57BL/6 mice and transduced with retroviral vectors encoding IRES-GFP (MIG) and SIGIRR-IRES-GFP (SIGIRR). Cells were cultured with antigen mBSA for 3 days and analyzed with flow cytometry following intracellular staining. **D** Relative expression levels of TNF-α mRNAs in retrovirally transduced memory CD4 T cells cultured in the presence of mBSA. Expression levels were monitored by quantitative real-time RT–PCR. The relative fold changes were calculated using the ΔΔCt method; data from all the samples were normalized to the SIGIRR group, and *ACTB* served as an endogenous control. **E** Knee joint swelling of *Sigirr* ^+*/*+^ (WT) and SIGIRR KO mice (KO) that received 500 μg of anti-TNF-α monoclonal antibody (clone XT3.11, Bio X Cell) or rat IgG1 κ isotype control antibody (Bio X Cell) intraperitoneally at days 0 and 3 during AIA. AIA was induced as described in the Materials and Fig. 3. Statistical significance was determined by multiple t tests using the Holm‒Sidak method, with alpha = 0.05 (* indicates significance at *P < 0.05 and *n* = 4)

To investigate whether exacerbated arthritis symptoms were caused by enhanced TNF-α expression in CD4 T cells upon SIGIRR deficiency, we examined cytokine production in memory CD4^+^ T cells upon antigen immunization. As described above (Fig. 3G), *Rag1*^*−/−*^ mice were transferred with *Sigirr* ^+*/*+^ or *Sigirr *^*−/−*^ naïve CD4 T cells and then immunized with CFA/mBSA. Naïve T cells underwent maturation into memory T cells upon immunization (Gray et al. 2018; Zhu et al. 2010; Alzabin and Williams 2011). Seven days later, memory CD4 T cells (CD4^+^ CD44^hi^) in the spleens of these transferred *Rag1*^*−/−*^ mice were flow-sorted and cultured in the presence of mBSA for 72 h for intracellular staining. We found that the percentage of memory CD4 T cells expressing TNF-α was significantly augmented in *Rag1*^*−/−*^ mice that transferred *Sigirr *^*−/−*^ CD4 T cells (1.6-fold) compared with *Sigirr* ^+*/*+^ CD4 T cells (Fig. 4A, B). However, there was a slight increase in IL-17-producing cells among memory CD4 T cells in the spleens of *Rag1*^*−/−*^ mice transferred with *Sigirr *^*−/−*^ CD4 T cells compared with those transferred with *Sigirr* ^+*/*+^ CD4 T cells (Fig. 4A, B). To confirm the restraint of SIGIRR on TNF-α expression in memory CD4 T cells, C57BL/6 mice were immunized with mBSA in CFA to harvest the largest number of antigen-experienced CD4 T cells as described in the Materials and Methods. After 7 days, memory CD4 T cells were flow-sorted from spleens from the immunized mice. We used retroviral transduction to deliver SIGIRR-IRES-EGFP to purified memory CD4 T cells and cultured them with mBSA for 3 days (Additional file 7: Figure S7). Overexpression of SIGIRR resulted in less induction of TNF-α in a fraction of the transduced (EGFP^+^) T cells from C57BL/6 wild-type mice in contrast to cells transduced with IRES-EGFP control vector (top right quadrants of scatter plot in Fig. 4C), while frequencies of TNF-producing EGFP^−^ cells (top left quadrants) in both groups were comparable, implying the drop of TNF-producing EGFP^+^ cells was caused by SIGIRR directly and exactly. Quantitative RT–PCR also indicated that the enforced expression of SIGIRR resulted in reduced transcription of TNF-α (Fig. 4D). To demonstrate the contribution of TNF-α to the development of AIA in *Sigirr *^*−/−*^ mice, *Sigirr* ^+*/*+^ and *Sigirr *^*−/−*^ mice were immunized with CFA/mBSA to induce an AIA model and then treated with neutralizing antibody against TNF-α or isotype antibody (Fig. 4E). We observed that *Sigirr *^*−/−*^ mice injected with the isotype antibody showed the greatest joint swelling compared to *Sigirr* ^+*/*+^ mice injected with the isotype antibody, consistent with the phenotype illustrated in Fig. 3A. Neutralizing antibody against TNF-α significantly ameliorated articular swelling both in *Sigirr* ^+*/*+^ and *Sigirr *^*−/−*^ mice compared with their littermates treated with isotype antibody. Importantly, *Sigirr *^*−/−*^ mice treated with neutralizing antibody against TNF-α retained a more severely swollen ankle than *Sigirr* ^+*/*+^ mice treated with the same antibody, emphasizing that the vulnerability to AIA in *Sigirr *^*−/−*^ mice was mediated by unleashed production of TNF-α to a large extent. Together, these data identified a defect of SIGIRR in memory CD4 T cells, leading to massive TNF-α production and exacerbating articular inflammation in mice.

### SIGIRR deficiency leads to increased IL-1β-induced C/EBPβ phosphorylation

**Fig. 5:**
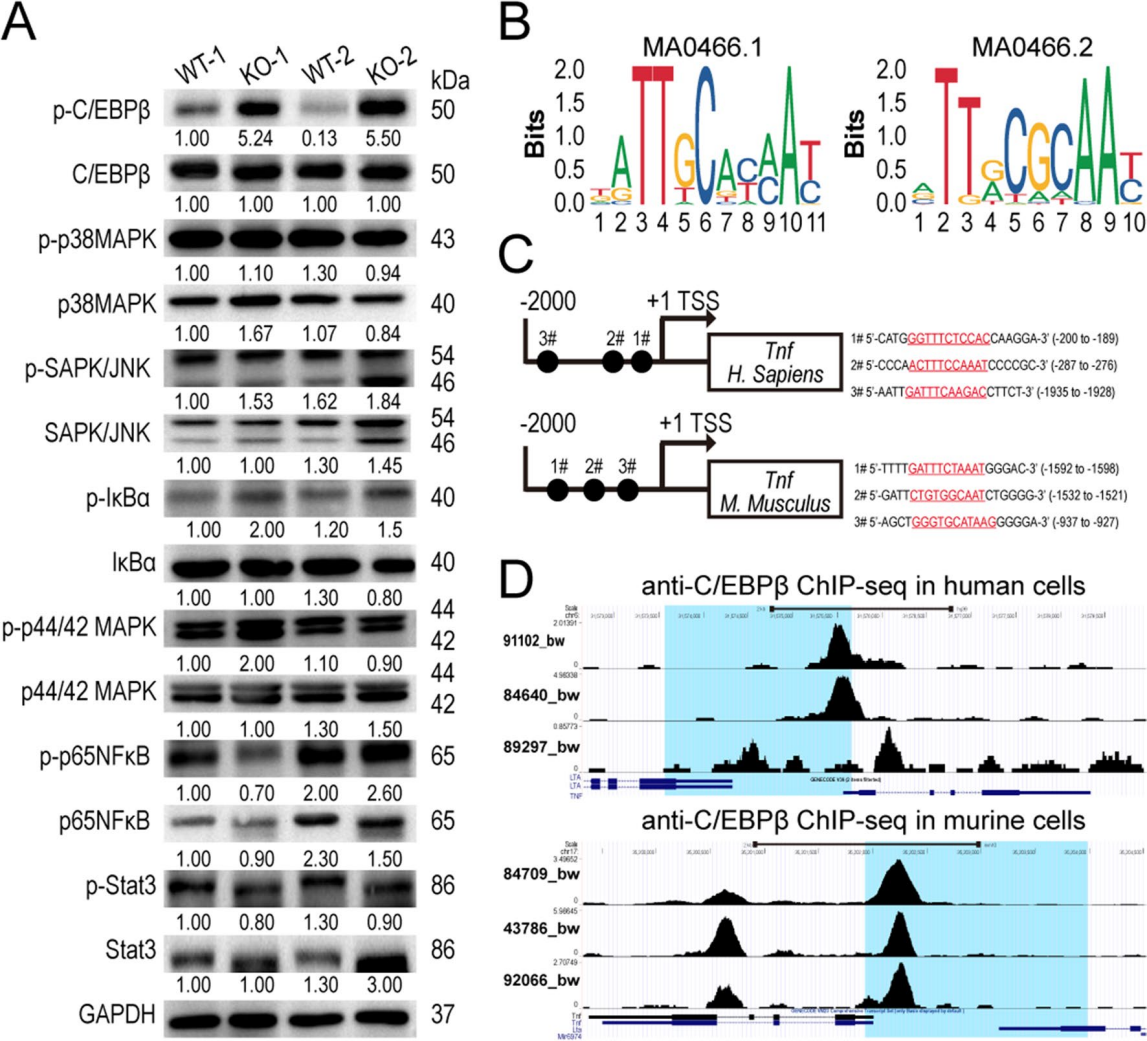
Enhanced activity of C/EBPβ in *Sigirr *^*−/−*^ CD4 T cells upon IL-1β stimulation. **A** Cell lysates from wild-type and *Sigirr *^*−/−*^ CD4 T cells (n = 2) treated with IL-1β (10 ng/ml) for 30 min were analyzed by SDS–PAGE followed by western blotting using antibodies for the indicated molecules. The quantification of each immunoblot is labeled under each band, and the molecular weight (kDa) is displayed on the right side. **B** Two classical binding motifs of transcription factor C/EBPβ notated by JASPER database. **C** Schematic representation of C/EBPβ binding sites for *TNF-α* promotor sequence (upper, homo sapiens and lower, mus musculus) and those binding site-containing nucleotide sequences are shown at right. **D** UCSC genome track view of C/EBPβ ChIP-seq signals in different loci of *TNF-α* genes and promotor regions, 2 kb nucleotides relative to the transcriptional start site of *TNF-α*, are shaded in blue. The upper panel shows the binding signal to Homo sapiens *TNF-α* genes in monocyte THP-1, leukemia cell MV4;11, epididymis epithelial cells. The lower panel displays the binding signal to Mus musculus *TNF-α* genes in macrophage MIN, microglial cell BV2 and bone marrow-derived macrophages. Raw ChIP-seq data and treatment protocols are available in the Gene Expression Omnibus (GEO) database (https://www.ncbi.nlm.nih.gov/geo/) under the accession numbers GSM3112102, GSM2345027, GSM1010802, GSM2663837, GSM1315484, and GSM2974847

**Fig. 6:**
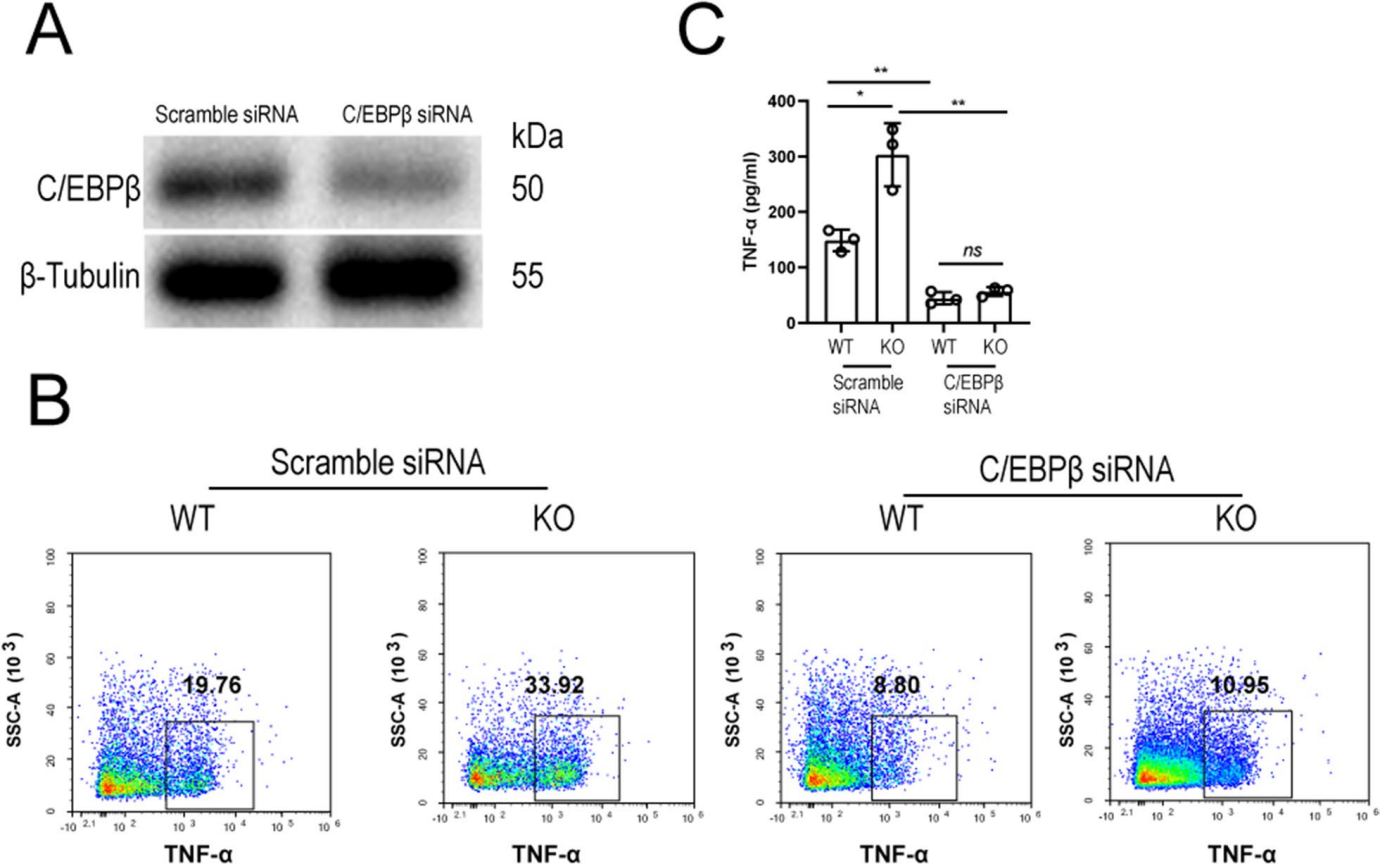
Increased TNF-α production led by SIGIRR deficiency was largely abolished by C/EBPβ gene knockdown in primary CD4 T cells treated with IL-1β. Primary CD4 T cells were isolated from wild-type or *Sigirr *^*−/−*^ mice spleens and transfected with control or targeting small interfering RNA (siRNA). After 3 days, the primary CD4 T cells were treated with IL-1β for 48 h. The supernatants were collected for ELISA, and the cells were collected for intracellular staining by flow cytometry. **A** C/EBPβ gene silencing in wild-type mouse primary CD4 T cells 3 days after transfection with control siRNA (scramble siRNA) or C/EBP β-specific siRNA (C/EBPβ siRNA). **B** Representative FACS plot of intracellular TNF-α expression in wild-type and *Sigirr *^*−/−*^ CD4 T cells transfected with scramble or C/EBPβ siRNA. **C** TNF-α production in the supernatants of wild-type and *Sigirr *^*−/−*^ CD4 T cells transfected with scramble or C/EBPβ siRNA. *ns*, not significant (*P* > 0.05), *P < 0.05, **P < 0.01 and ***P < 0.001 (unpaired t test, n = 3)

To understand the molecular mechanism by which SIGIRR regulates TNF-α production in CD4 T cells during AIA development, splenic CD4 T cells after the first immunization were isolated for phosphorylation of the TIR downstream signaling cascade. However, phosphorylation of the classic TIR signaling components p38MAPK, JNK and p44/42 MAPK was not altered in *Sigirr *^*−/−*^ CD4 T cells compared to WT cells (Fig. 5A). Additionally, the activities of IκBα, NK-κB and STAT3 did not seem to be significant in our study (Fig. 5A). Hence, we shifted our focus from the activities of those common transcription factors to some essential and RA-specific ones (Zhou et al. 2011; Tsushima et al. 2015; Ha et al. 2016; Takeuchi and Akira 2010; Hacker et al. 2006). Here, we found that phosphorylation of the regulatory transcription factor C/EBPβ was increased in *Sigirr *^*−/−*^ CD4 T cells (Fig. 5A). C/EBPβ has been shown to activate *TNF-α* gene transcription in myelomonocytic cells or T cells by binding to specific sites of *TNF-α* promoter sequence directly. We then analyzed the *TNF-α* promoter using the JASPAR database for prediction of potential C/EBPβ binding sites. As shown in Fig. 5B and C, both the human and murine *TNF-α* genes comprise two putative C/EBPβ consensus motif (MA0466.1 and MA0466.2) binding sites. Cistrome data browser retrieving showed genuine binding of C/EBPβ to specific nucleotide sequence upstream of *TNF-α* transcript in human monocytes or mouse macrophages (Fig. 5D), consistent with the predicted binding sites demonstrated in Fig. 5C. To validate the contribution of C/EBPβ signaling to upregulated TNF-α production in *Sigirr *^*−/−*^ CD4 T cells, we performed a loss-of-function experiment for C/EBPβ with transfection using small interfering RNA (siRNA, Fig. 6A). We found that the increased TNF-α production caused by SIGIRR deficiency was abrogated to a large extent (siRNA, Fig. 6B, C), highlighting the essential roles of C/EBPβ signaling in enhanced TNF-α production in *Sigirr *^*−/−*^ CD4 T cells. Thus, SIGIRR preferentially affected IL-1β-mediated C/EBPβ activation rather than other MAPK member or NF-κB, to suppress TNF-α production in CD4 T cells, explained by experimental evidence and cis-acting factor bioinformatics analysis.

## Discussion

In this study, we combined single-cell RNA-sequencing analysis, genetic approaches and intervention strategies to describe a fundamental suppression of SIGIRR on the C/EBPβ/TNF-α signaling axis in orchestrating rheumatoid inflammation in patients with RA and a monoarticular model of experimental arthritis in memory CD4 T cells.

SIGIRR deficiency was associated with exacerbated inflammatory and immune reactions under a variety of conditions (Molgora et al. 2017). Previous studies have demonstrated that SIGIRR influences chronic inflammatory or autoimmune diseases, such as intestinal inflammation, systemic lupus erythematosus, and psoriatic inflammation, by regulating the activities of NK cells, CD8 T cells or γδ T cells (Wang et al. 2022; Wang et al. 2013; Molgora et al. 2017; Xiao et al. 2007; Russell et al. 2013). Memory CD4 T cells are of great interest in the context of autoimmune diseases because of their long-lived nature, efficient responses to antigens, and unique potential to mediate recurring autoimmune responses (Raphael et al. 2020). Based on the relatively higher expression of the *SIGIRR* gene on human memory CD4 T cells or CD4 T cells predicted by single-cell or bulk transcriptomic analysis, memory CD4 T cells were focused on demonstrating the functions of SIGIRR in RA, although gene polymorphisms and the regulation of SIGIRR in rheumatoid arthritis have been reported in earlier studies (Drexler et al. 2010; Yang et al. 2021). We observed an aberrant downregulation of SIGIRR and enhanced TNF-α production in memory CD4 T cells from the peripheral blood of RA patients and found that SIGIRR deficiency promoted the progression of AIA mediated by elevated TNF-α expression, thus establishing a closer association between SIGIRR and TNF-α production in memory CD4 T cells in RA for the first time. It was reported that loss of N-linked glycosylation on SIGIRR in colorectal cancer blocked the localization of full-length SIGIRR to the surface of colon epithelial cells and its ability to downregulate IL1R signaling (Zhao et al. 2015). It could be possible that SIGIRR dysfunction also existed in RA CD4 T cells, which is worth investigating in the future.

TNF is a major cause of the excess inflammation that drives RA (Kalliolias and Ivashkiv 2016), and it is likely to remain the preferred target of first-line biologic therapy for the foreseeable future, as in populations with active RA despite ongoing, nonbiologic, DMARD therapy (Emery et al. 2014; Taylor and Feldmann 2009). In addition to monocytes, lymphocytes, including T cell and B cell populations, made a nonnegligible contribution to TNF production, as revealed by single-cell transcriptomics analysis of a large collection of human RA synovial tissue (Zhang et al. 2019). In the present study, SIGIRR^+^ memory CD4 T cells exhibited much greater downregulation of TNF-α production than SIGIRR^−^ memory CD4 T cells. At the same time, enforced *SIGIRR* expression in RA SIGIRR^−^ memory CD4 T cells reversed the production of TNF-α, echoing the downregulated TNF-α production in RA SIGIRR^+^ memory CD4 T cells. Subsequently, the suppressive roles of SIGIRR on TNF-α production were confirmed by CD4 T cells transfer experiments i*n vivo* and ectopic expression of SIGIRR in memory CD4 T cells ex vivo. Neutralizing antibody against TNF-α in vivo underlined that the vulnerability to AIA in *Sigirr *^*−/−*^ mice was largely mediated by unleashed production of TNF-α. In addition, the effect of SIGIRR on the production of IL-17 in CD4 T cells was also considered; however, this effect was marginal, unlike a previous report (Gulen et al. 2010), probably due to the different stimulation procedures used in our study.

We explored the molecular mechanisms for soaring TNF-α production in SIGIRR-deficient CD4 T cells and found that this could be attributed to hyperphosphorylation of C/EBPβ elicited by the absence of SIGIRR, supported by experimental evidence and cis-acting factor bioinformatics analysis. As an unconventional transcription factor in TIR signaling, C/EBPβ and its target genes (*TNF, Csf3, S100a8*) were intimately linked to innate immune cell expansion, and host factor-derived inflammation in RA may account for, at least in part, the proinflammatory cytokine networks and tissue-damaging cellular activities in synovitis observed in SIGIRR-deficient mice. The molecular associations of SIGIRR interfering TIR signaling have been investigated extensively (Qin et al. 2005; Gulen et al. 2010), and the precious physical events of the SIGIRR/C/EBPβ/TNF-α signaling axis will be revealed in the future.

In summary, our results allowed us to propose and provide evidence to support the SIGIRR/C/EBPβ/TNF-α signaling axis hypothesis in memory CD4 T cells in RA, raising the possibility that receptor reversion can target key abnormalities in T cells and thus representing a potentially novel strategy for immunomodulatory therapy in T cell -mediated autoimmune diseases.

## Conclusions

Our present study demonstrated that SIGIRR deficiency contributed to memory CD4 T-cell abnormalities by facilitating the IL1/C/EBPβ/TNF-α signaling axis in rheumatoid arthritis, highlighting a novel molecular mechanism for RA development and the potential application of SIGIRR activation for RA treatment.

## Supplementary Information

**Additional file 1: Figure S1.** Feature plots for various genes that help to attribute identities. The intensity of blue is related to the relative expression of the gene in a particular cell.

**Additional file 2: Figure S2.** Frequency of SIGIRR in memory CD4 T cells in PBMCs of RA patients stratified by CCP expression.

**Additional file 3: Figure S3.** Cytokine production in SIGIRR-positive or SIGIRR-negative memory CD4 T cells of patients with RA and the healthy population. Related to Fig. 2. (A-B), Memory CD4 T cells were identified as CD4 ^+^ CD45RO ^+^ CD45RA ^−^ previously gated on single live (FVS780 ^−^) lymphocytes (FSC ^low^ SSC ^low^). Representative flow cytometry contour plots for intracellular cytokine production among SIGIRR^+^ memory CD4 T cells in healthy and RA cohorts (n = 50 for healthy and 78 for RA individuals), with quantification of results as frequency (B). (C), SIGIRR^+^ and SIGIRR^−^ memory CD4^+^ T cells of healthy or RA patients were flow-sorted and seeded in 96-well cell culture plates and then treated with IL-1β in the presence of α-CD3/α-CD28 antibodies. After 48 h, the supernatants were collected for TNF-α production measurement by ELISA. *ns*, not significant (*P* > 0.05) and ***P < 0.001 (unpaired t test, n = 13 and 10 for healthy and RA memory CD4 T cells).

**Additional file 4: Figure S4.** Targeted disruption of the mouse gene encoding SIGIRR. (A), Schematic of *Sigirr* deletion in C57BL/6 mice and primers for genotyping. The offspring were born at the expected Mendelian ratios. (B), *Sigirr* genotyping using the F1, F2 and R1 primers listed in the Materials and Methods. The red arrow shows the knockout band (700 bp), and the blue arrow shows the wild-type band (500 bp). (C), Splenomegaly in naïve SIGIRR KO mice and the ratios of spleen weight to body weight (g/g) in mice of *Sigirr* ^+/+^ and *Sigirr *^−/−^genotypes.

**Additional file 5: Figure S5.** Composition of splenic immune cells and CD4^+^ T cell subpopulations in healthy *Sigirr* ^+/+^ mice and *Sigirr *^−/−^ mice. (A), Frequencies of major immune populations in spleens between *Sigirr* ^+/+^ mice (n = 5) and *Sigirr *^−/−^ mice (n = 5). (B), Absolute number and frequency of granulocytes among 2.5 × 10^5^ live CD45^+^ leukocytes *derived from Sigirr* ^+/+^ and *Sigirr *^−/−^mice. (C), Representative plots of T cells (CD3^+^ NK1.1^−^), NK cells (CD3^−^ NK1.1^+^), NKT cells (CD3^+^ NK1.1^+^), B cells (CD3^−^ CD19^+^), CD4 T cells (CD3^+^ CD4^+^ CD8^−^), CD8 T cells (CD3^+^ CD4^−^ CD8^+^), CD11b^+^ Gr1^hi^ granulocytes and CD11b^+^ Gr1^hi^ mononuclear phagocytes among live CD45^+^ leukocytes in *Sigirr* ^+/+^ and *Sigirr *^−/−^mice spleens. (D), Bar graphs representing absolute numbers (left) and frequency (right) of subpopulations among live CD45^+^ leukocytes as measured by flow cytometry in the spleens of *Sigirr* ^+/+^ and *Sigirr *^−/−^mice. (E–F), Representative plots of naïve (CD62L^+^ CD44^−^), effector (CD62L^+^ CD44^−^), CD62L^+^ CD44^+^ cells and regulatory T cells (CD25^+^, F) among live CD4 T cells. (G) Absolute number (upper) and frequency (lower) of naïve, effector, CD62L^+^ CD44^+^ cells and regulatory T cells among CD4^+^ T cells in the spleens of *Sigirr* ^+/+^ and *Sigirr *^−/−^mice. (H) Ratio of effector to naïve CD4^+^ T cells (E/N) between *Sigirr* ^+/+^ mice (n = 5) and *Sigirr *^−/−^mice (n = 5). *ns*, not significant (*P* > 0.05), *P < 0.05, **P < 0.01 and ***P < 0.001 and ****P < 0.0001 (unpaired t test).

**Additional file 6: Figure S6.** Gating strategy used to sort naïve CD4 T cells by flow cytometry. After performing cell surface staining with anti-CD4, CD25, CD62L and CD44 antibodies conjugated with fluorescein, naïve CD4 T cells (After) were sorted from splenic suspensions from Sigirr ^+*/*+^ *mice* (Before) according to the CD4^+^ CD25^−^ CD62L^+^ CD44^−^ strategy among live lymphocytes.

**Additional file 7: Figure S7.** Ectopic expression of SIGIRR in purified CD4^+^ T cells after transfection with retrovirus packaged by phoenix helper-free retrovirus producer lines. The expression of SIGIRR (50 kDa) and GFP (27 kDa) in CD4 T-cell lysates was detected by immunoblotting with GAPDH as a loading control.

**Additional file 8: Table S1.** Marker Genes used for Cluster Annotation in Single Cell RNA-seq Analysis.

**Additional file 9: Table S2.** The Gating Scheme of Isolations of Cells from Whole Blood or PBMCs.

**Additional file 10: Table S3.** Characteristics of Rheumatoid Arthritis Patients and Healthy Cohorts.

**Additional file 11: Table S4.** Fluorescence antibody used for flow cytometry and sorting.

**Additional file 12: Table S5.** Primer Sets for qRT‒PCR and Mouse Genotyping.

**Additional file 13: Table S6.** Antibodies Used for Immunoblots.

## Data Availability

The data and materials of this study are available from the corresponding authors upon request.

## Abbreviations

SIGIRR: Single immunoglobulin IL-1R-related receptor
C/EBPβ: CCAAT/enhancer binding protein beta
RA: Rheumatoid arthritis
AIA: Antigen-induced arthritis
mBSA: Methylated bovine serum albumin
TNF: Tumor necrosis factor
