# Supplementary figures and images for: SIGIRR deficiency contributes to CD4 T cell abnormalities by facilitating the IL1/C/EBPβ/TNF-α signaling axis in rheumatoid arthritis
Source: Mol Med. 2022 Nov 18;28:135. doi: 10.1186/s10020-022-00563-9 (PMC9673409; doi:10.1186/s10020-022-00563-9)

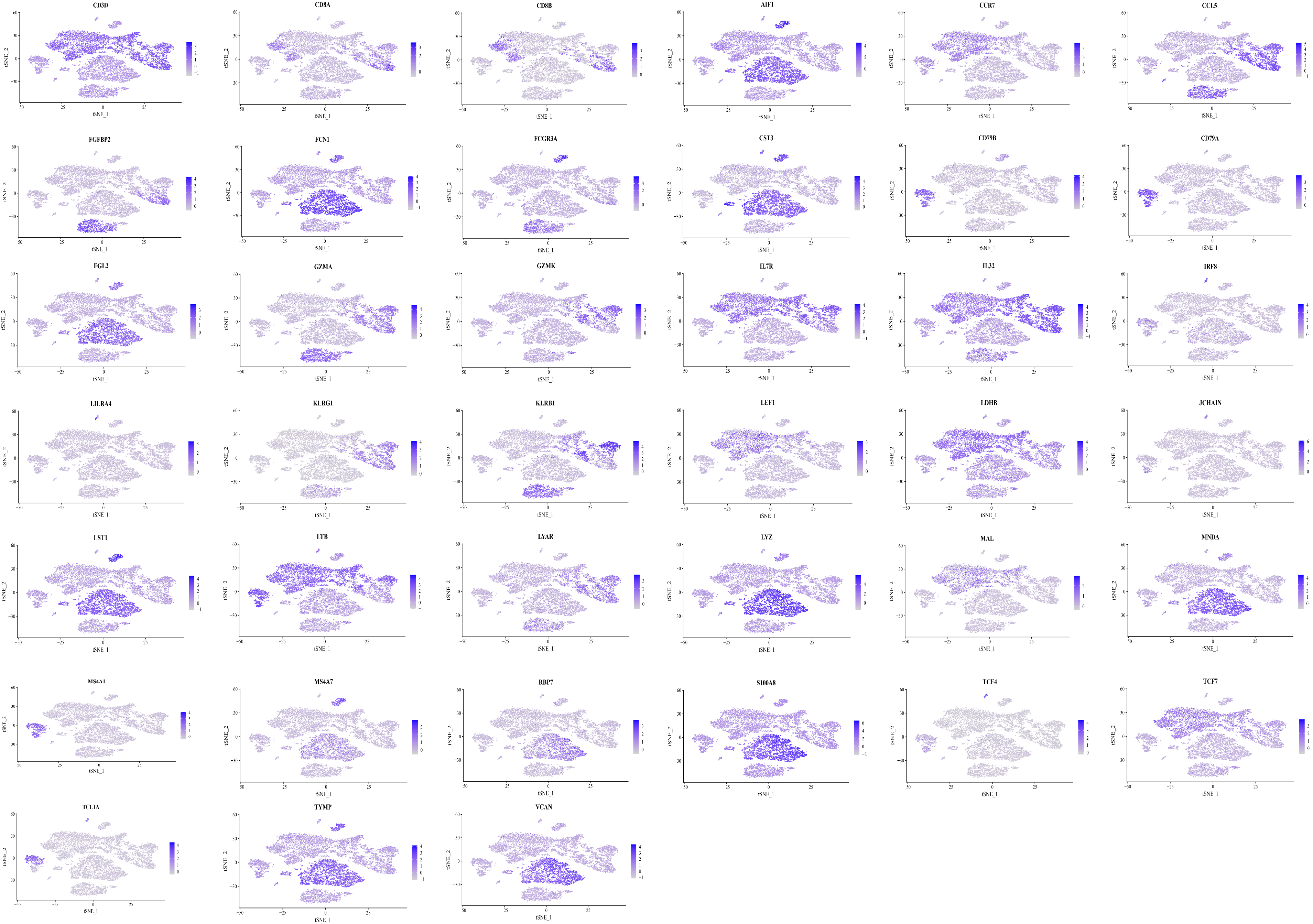

Supplement: Supplementary file 1 — Additional file 1: Figure S1. Feature plots for various genes that help to attribute identities. The intensity of blue is related to the relative expression of the gene in a particular cell. [file 10020_2022_563_MOESM1_ESM.tif]

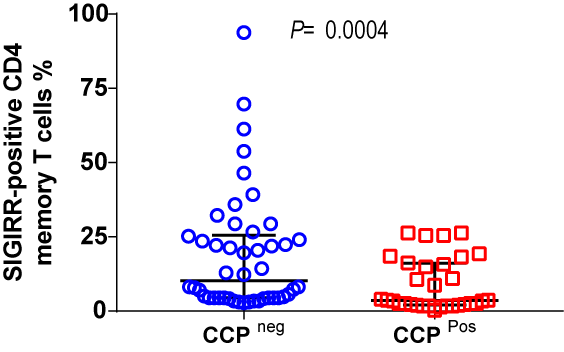

Supplement: Supplementary file 2 — Additional file 2: Figure S2. Frequency of SIGIRR in memory CD4 T cells in PBMCs of RA patients stratified by CCP expression. [file 10020_2022_563_MOESM2_ESM.tif]

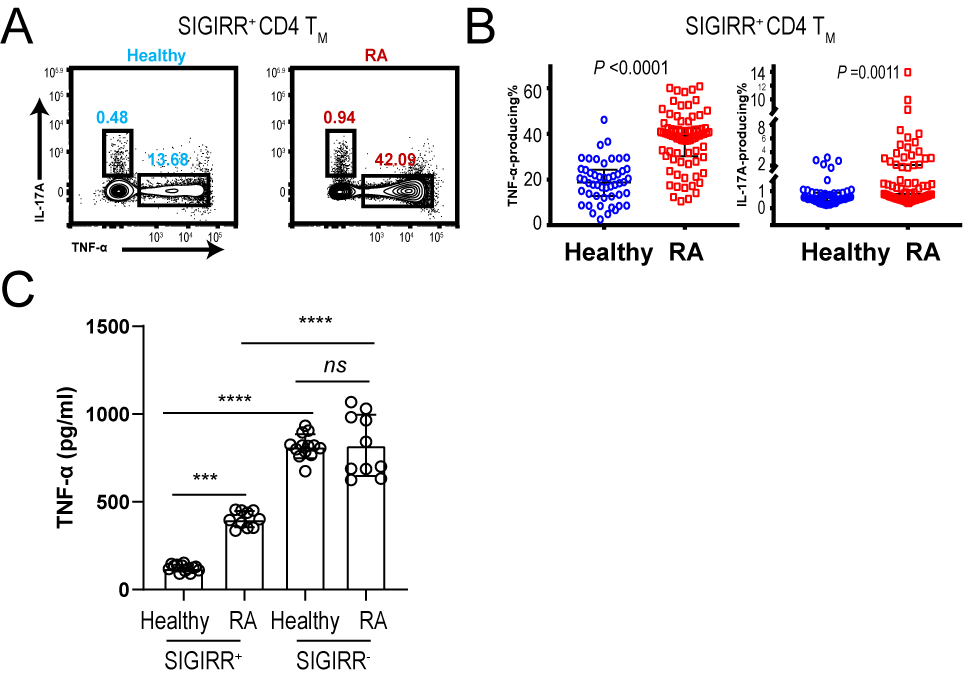

Supplement: Supplementary file 3 — Additional file 3: Figure S3. Cytokine production in SIGIRR-positive or SIGIRR-negative memory CD4 T cells of patients with RA and the healthy population. Related to Fig. 2. (A-B), Memory CD4 T cells were identified as CD4 + CD45RO + CD45RA − previously gated on single live (FVS780 −) lymphocytes (FSC low SSC low). Representative flow cytometry contour plots for intracellular cytokine production among SIGIRR+ memory CD4 T cells in healthy and RA cohorts (n = 50 for healthy and 78 for RA individuals), with quantification of results as frequency (B). (C), SIGIRR+ and SIGIRR− memory CD4+ T cells of healthy or RA patients were flow-sorted and seeded in 96-well cell culture plates and then treated with IL-1β in the presence of α-CD3/α-CD28 antibodies. After 48 h, the supernatants were collected for TNF-α production measurement by ELISA. ns, not significant (P > 0.05) and ***P < 0.001 (unpaired t test, n = 13 and 10 for healthy and RA memory CD4 T cells). [file 10020_2022_563_MOESM3_ESM.tif]

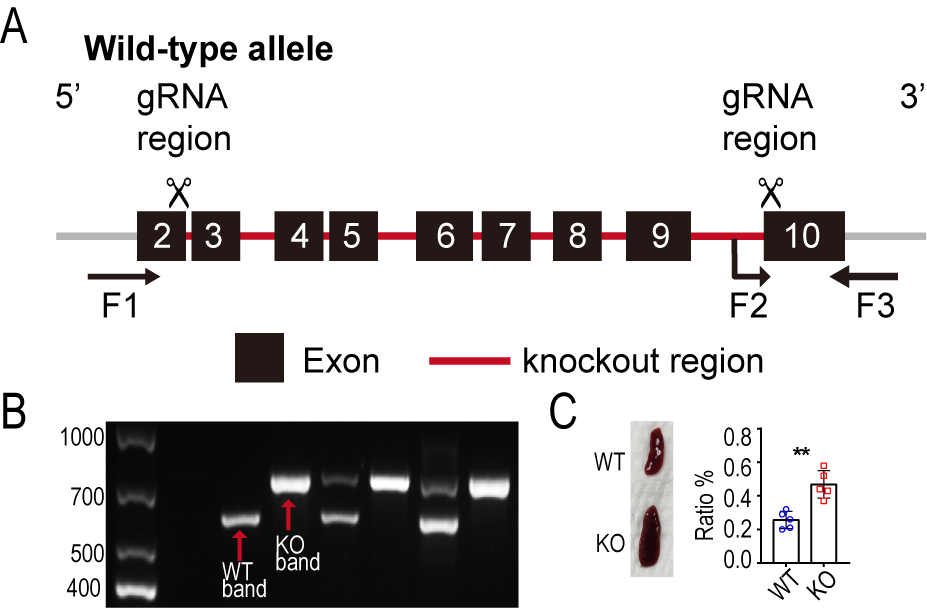

Supplement: Supplementary file 4 — Additional file 4: Figure S4. Targeted disruption of the mouse gene encoding SIGIRR. (A), Schematic of Sigirr deletion in C57BL/6 mice and primers for genotyping. The offspring were born at the expected Mendelian ratios. (B), Sigirr genotyping using the F1, F2 and R1 primers listed in the Materials and Methods. The red arrow shows the knockout band (700 bp), and the blue arrow shows the wild-type band (500 bp). (C), Splenomegaly in naïve SIGIRR KO mice and the ratios of spleen weight to body weight (g/g) in mice of Sigirr +/+ and Sigirr −/−genotypes. [file 10020_2022_563_MOESM4_ESM.tif]

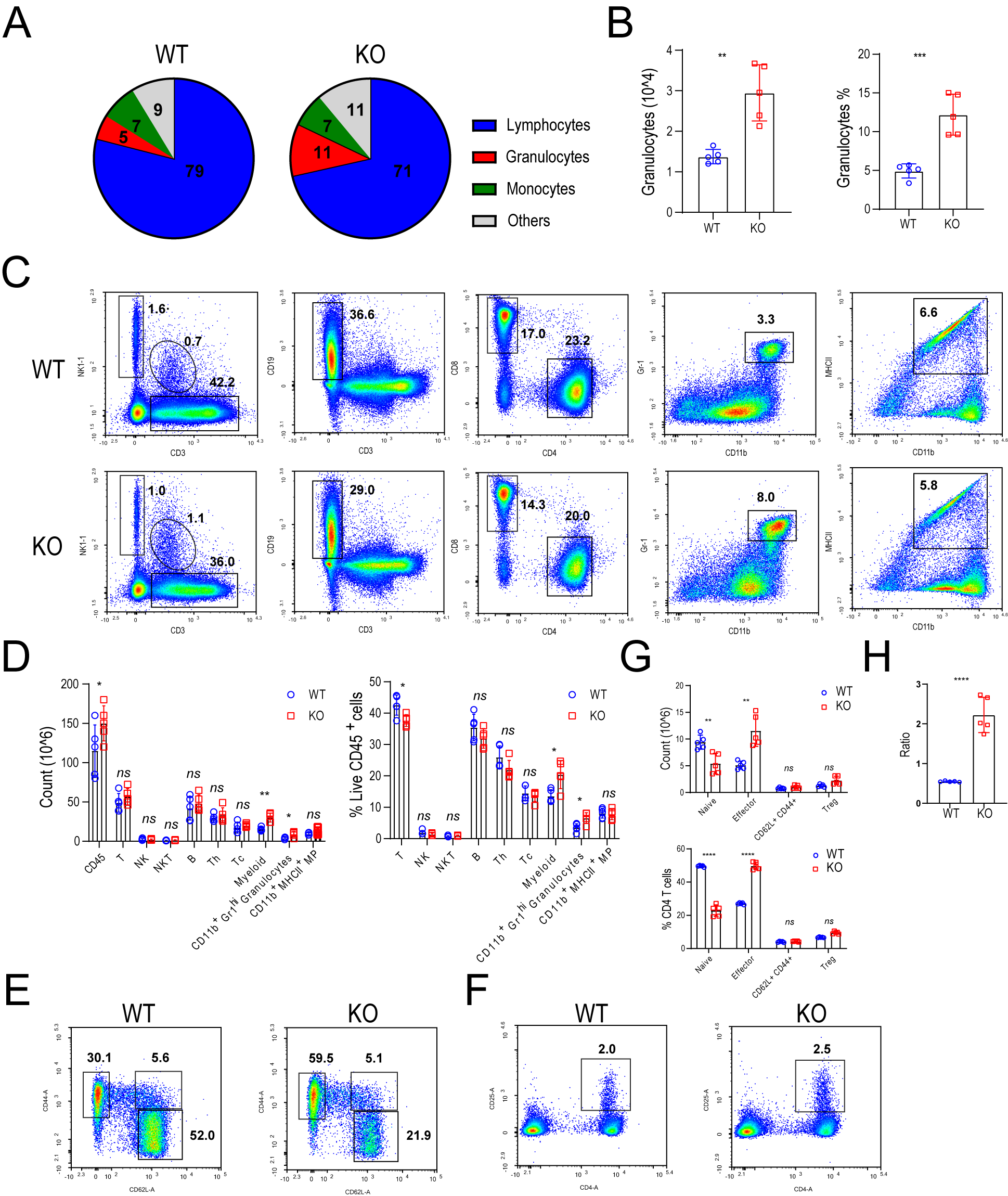

Supplement: Supplementary file 5 — Additional file 5: Figure S5. Composition of splenic immune cells and CD4+ T cell subpopulations in healthy Sigirr +/+ mice and Sigirr −/− mice. (A), Frequencies of major immune populations in spleens between Sigirr +/+ mice (n = 5) and Sigirr −/− mice (n = 5). (B), Absolute number and frequency of granulocytes among 2.5 × 10^5^ live CD45+ leukocytes derived from Sigirr +/+ and Sigirr −/−mice. (C), Representative plots of T cells (CD3+ NK1.1−), NK cells (CD3− NK1.1+), NKT cells (CD3+ NK1.1+), B cells (CD3− CD19+), CD4 T cells (CD3+ CD4+ CD8−), CD8 T cells (CD3+ CD4− CD8+), CD11b+ Gr1hi granulocytes and CD11b+ Gr1hi mononuclear phagocytes among live CD45+ leukocytes in Sigirr +/+ and Sigirr −/−mice spleens. (D), Bar graphs representing absolute numbers (left) and frequency (right) of subpopulations among live CD45+ leukocytes as measured by flow cytometry in the spleens of Sigirr +/+ and Sigirr −/−mice. (E–F), Representative plots of naïve (CD62L+ CD44−), effector (CD62L+ CD44−), CD62L+ CD44+ cells and regulatory T cells (CD25+, F) among live CD4 T cells. (G) Absolute number (upper) and frequency (lower) of naïve, effector, CD62L+ CD44+ cells and regulatory T cells among CD4+ T cells in the spleens of Sigirr +/+ and Sigirr −/−mice. (H) Ratio of effector to naïve CD4+ T cells (E/N) between Sigirr +/+ mice (n = 5) and Sigirr −/−mice (n = 5). ns, not significant (P > 0.05), *P < 0.05, **P < 0.01 and ***P < 0.001 and ****P < 0.0001 (unpaired t test). [file 10020_2022_563_MOESM5_ESM.tif]

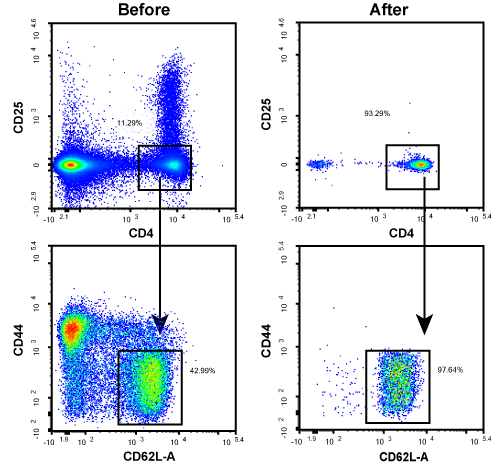

Supplement: Supplementary file 6 — Additional file 6: Figure S6. Gating strategy used to sort naïve CD4 T cells by flow cytometry. After performing cell surface staining with anti-CD4, CD25, CD62L and CD44 antibodies conjugated with fluorescein, naïve CD4 T cells (After) were sorted from splenic suspensions from Sigirr +/+ mice (Before) according to the CD4+ CD25− CD62L+ CD44− strategy among live lymphocytes. [file 10020_2022_563_MOESM6_ESM.tif]

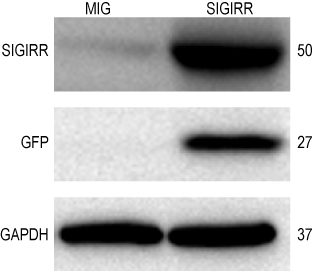

Supplement: Supplementary file 7 — Additional file 7: Figure S7. Ectopic expression of SIGIRR in purified CD4+ T cells after transfection with retrovirus packaged by phoenix helper-free retrovirus producer lines. The expression of SIGIRR (50 kDa) and GFP (27 kDa) in CD4 T-cell lysates was detected by immunoblotting with GAPDH as a loading control. [file 10020_2022_563_MOESM7_ESM.tif]
