# Supplementary material for: SIGIRR deficiency contributes to CD4 T cell abnormalities by facilitating the IL1/C/EBPβ/TNF-α signaling axis in rheumatoid arthritis
Source: Mol Med. 2022 Nov 18;28:135. doi: 10.1186/s10020-022-00563-9 (PMC9673409; doi:10.1186/s10020-022-00563-9)
Supplement: Supplementary file 8 — Additional file 8: Table S1. Marker Genes used for Cluster Annotation in Single Cell RNA-seq Analysis. [file 10020_2022_563_MOESM8_ESM.pdf]

Cluster ID	Markers	Cell Type
c0	*TCF7, MAL, LEF1*	CD4 TN1
c1	*S100A8, LYZ, VCAN, S100A9, MNDA*	Mono1
c2	*CCR7, TCF7, IL7R, LEF1*	CD4 TN2
c3	*GNLY, NKG7, FGFBP2, GZMB*	NK1
c4	*S100A8, S100A9, S100A12*	Mono2
c5	*IL32, LTB, IL7R, CD3D*	CD4 Treg
c6	*GZMH, CCL5, CD8A, FGFBP2*	CD8 TM
c7	*CST3, FCN1, FGL2, AIF1, TYMP*	Mono3/4
c8	*KLRG1, CCL5, LYAR, IL7R*	CD4 TM1
c9	*TCL1A, CD79A, MS4A1, CD79B*	B cells1
c10	*CD8B, TCF7, LEF1, IL7R, CD8A*	CD8 TN
c11	*KLRB1, KLRG1, GZMK, GZMA, IL7R*	CD4 TM2
c12	*CCL5, IL32, GZMK, KLRB1, KLRG1*	CD4 TM3
c13	*FCGR3A, LST1, AIF1, SAT1, MS4A7*	DC4 ($CD1C^{-}$ $CD141^{-}$ $CD11C^{+}$)
c14	*BANK1, MS4A1, CD79A*	B cells2
c15	*PF4, PPBP, GNG11*	Platelet
c16	*FCER1A, CD74, HLA-DRB5, HLA-DQA1*	Macrophage
c17	*KLRC1, GNLY, KLRD1, XCL2*	NK2
c18	*LILRA4, JCHAIN, TCF4, IRF8*	DC6 (pDC)
