# Supplementary material for: SIGIRR deficiency contributes to CD4 T cell abnormalities by facilitating the IL1/C/EBPβ/TNF-α signaling axis in rheumatoid arthritis
Source: Mol Med. 2022 Nov 18;28:135. doi: 10.1186/s10020-022-00563-9 (PMC9673409; doi:10.1186/s10020-022-00563-9)
Supplement: Supplementary file 9 — Additional file 9: Table S2. The Gating Scheme of Isolations of Cells from Whole Blood or PBMCs. [file 10020_2022_563_MOESM9_ESM.pdf]

| Gating Scheme of Isolations of Cells from Whole Blood or PBMC (cited from Uhlen-*SCIENCE*-2019) | | |
|---|---|---|
| Granulocytes (whole bood-derived) | Neutrophil | single CD45$^{+}$ SSC$^{hi}$ CD15$^{+}$ CD16$^{+}$ cells |
| | Eosinophil | single CD45$^{+}$ SSC$^{hi}$ CD15$^{lo}$ CD16$^{lo}$ HLA-Dr$^{-}$ CD193$^{+}$ cells |
| | Basophil | single CD45$^{+}$ SSC$^{hi}$ CD15$^{lo}$ CD16$^{lo}$ CD38$^{+}$ CD203c$^{+}$ HLA-Dr$^{-}$ CD193$^{+}$ CD123$^{+}$ cells |
| B cells (PBMC-derived) | Naïve | single SSC$^{lo}$ CD14$^{-}$ CD19$^{+}$ CD3$^{-}$ CD27$^{-}$ cells |
| | Memory | single SSC$^{lo}$ CD14$^{-}$ CD19$^{+}$ CD3$^{-}$ CD27$^{+}$ cells |
| NK cells (PBMC-derived) | / | single SSC$^{lo}$ CD14$^{-}$ CD19$^{+}$ CD3$^{-}$ CD56$^{+}$ cells |
| T cells (PBMC-derived) | Naïve CD4 | single SSC$^{lo}$ CD14$^{-}$ CD19$^{-}$ CD3$^{+}$CD4$^{+}$CD45RA$^{+}$ cells |
| | Memory CD4 | single SSC$^{lo}$ CD14$^{-}$ CD19$^{-}$ CD3$^{+}$CD4$^{+}$CD45RA$^{-}$ cells |
| | Regulatory CD4 | single SSC$^{lo}$ CD14$^{-}$ CD19$^{-}$ CD3$^{+}$CD4$^{+}$CD25$^{+}$ CD127$^{lo}$ CCR4$^{+}$ cells |
| | Naïve CD8 | single SSC$^{lo}$ CD14$^{-}$ CD19$^{-}$ CD3$^{+}$CD4$^{-}$ CD8$^{+}$CD45RA$^{+}$ cells |
| | Memory CD8 | single SSC$^{lo}$ CD14$^{-}$ CD19$^{-}$ CD3$^{+}$CD4$^{-}$ CD8$^{+}$CD45RA$^{-}$ cells |
| | γδT | single SSC$^{lo}$ CD14$^{-}$ CD19$^{-}$ CD3$^{+}$γδTCR$^{+}$ TCR Vα7.2$^{-}$ cells |
| | MAIT | single SSC$^{lo}$ CD14- CD19- CD3+γδTCR- TCR Vα7.2+ CD161+ cells |
| Monocytes (PBMC-derived) | Classic | single SSC$^{int}$ CD3$^{-}$ CD19$^{-}$ CD56$^{-}$ CD20$^{-}$ CD16$^{-}$ CD14$^{+}$ cells |
| | Non-classical | single SSC$^{int}$ CD3$^{-}$ CD19$^{-}$ CD56$^{-}$ CD20$^{-}$ CD16$^{+}$ CD14$^{-}$ cells |
| | Intermediate | single SSC$^{int}$ CD3$^{-}$ CD19$^{-}$ CD56$^{-}$ CD20$^{-}$ CD16$^{+}$ CD14$^{+}$ cells |
| Dendritic cells (PBMC-derived) | Plasmacytoid DC | single SSC$^{int}$ CD19$^{-}$ CD3$^{-}$ CD20$^{-}$ CD56$^{-}$ CD123$^{+}$ HLA-Dr$^{-}$ CD11c$^{-}$cells |
| | Myeloid DC | single SSC$^{int}$ CD19$^{-}$ CD3$^{-}$ CD14$^{lo}$ CD16$^{-}$ HLA-Dr$^{-}$ CD11c$^{+}$cells |
