# Supplementary material for: SIGIRR deficiency contributes to CD4 T cell abnormalities by facilitating the IL1/C/EBPβ/TNF-α signaling axis in rheumatoid arthritis
Source: Mol Med. 2022 Nov 18;28:135. doi: 10.1186/s10020-022-00563-9 (PMC9673409; doi:10.1186/s10020-022-00563-9)
Supplement: Supplementary file 10 — Additional file 10: Table S3. Characteristics of Rheumatoid Arthritis Patients and Healthy Cohorts. [file 10020_2022_563_MOESM10_ESM.pdf]

Supplemental Table 3 Characteristics of Rheumatoid Arthritis Patients and Healthy Cohorts

Characteristic	RA patients (n=78)	Healthy (n=50)
Age — yr	50.41±10.48	54.84±9.95
Female sex — no. (%)	59 (75.6)	35 (70.0)
ESR — mm/hr	22±22	—
Positive for C-reactive protein — no. (%)	43 (55.1)	—
Positive for CCP — no. (%)	32 (41.0)	—
Positive for rheumatoid factor — no. (%)	50 (64.1)	—
Tender-joint count	9.21±8.06	—
Swollen-joint count	6.40±6.33	—
Total-joint count	15.60±13.08	—
HAQ score	10.04±12.40	—
DAS28 score	4.72±1.52	—
