# Supplementary material for: SIGIRR deficiency contributes to CD4 T cell abnormalities by facilitating the IL1/C/EBPβ/TNF-α signaling axis in rheumatoid arthritis
Source: Mol Med. 2022 Nov 18;28:135. doi: 10.1186/s10020-022-00563-9 (PMC9673409; doi:10.1186/s10020-022-00563-9)
Supplement: Supplementary file 11 — Additional file 11: Table S4. Fluorescence antibody used for flow cytometry and sorting. [file 10020_2022_563_MOESM11_ESM.pdf]

Supplemental Table 4 Fluorescence antibody used for flow cytometry and sorting				
**Fluorochrome**	**Antigen**	**Clone**	**Supplier**	**Category**
FVS780	Cell surface and intracellular amines	/	BD Biosciences	discrimination of live/dead cells
FITC	CD4	RPA-T4	BioLegend	surface
PE/Cyanine7	CD45RA	HI100	BioLegend	surface
APC	CD45RO	UCHL1	BioLegend	surface
PE	SIGIRR	Clone #010	Sino Biological	surface
BUV395	TNF-α	MAB11	BD Biosciences	intracellular
BV421	IL-17A	N49-653	BD Biosciences	intracellular
BV510	CD45	30-F11	BD Biosciences	surface
APC	CD11b	M1/70	BD Biosciences	surface
FITC	Gr-1	RB6-8C5	BioLegend	surface
PE	F4/80	T45-2342	BD Biosciences	surface
Alexa Fluor 647	TNF-α	MP6-XT22	BioLegend	intracellular
PE/Cyanine7	IL-17A	TC11-18H10.1	BioLegend	intracellular
PE	CD45	30-F11	BioLegend	surface
BUV395	CD3	145-2C11	BD Biosciences	surface
PE/Cyanine7	CD4	GK1.5	BioLegend	surface
BV711	CD8	53-6.7	BioLegend	surface
BUV395	NK1.1	PK136	BD Biosciences	surface
APC-R700	CD19	1D3	BD Biosciences	surface
BV510	CD11b	M1/70	BD Biosciences	surface
FITC	Gr1	RB6-8C5	BioLegend	surface
BV421	I-A/I-E (MHCII)	M5/114.15.2	BD Biosciences	surface
