# Supplementary material for: SIGIRR deficiency contributes to CD4 T cell abnormalities by facilitating the IL1/C/EBPβ/TNF-α signaling axis in rheumatoid arthritis
Source: Mol Med. 2022 Nov 18;28:135. doi: 10.1186/s10020-022-00563-9 (PMC9673409; doi:10.1186/s10020-022-00563-9)
Supplement: Supplementary file 12 — Additional file 12: Table S5. Primer Sets for qRT‒PCR and Mouse Genotyping. [file 10020_2022_563_MOESM12_ESM.pdf]

**Supplemental Table 5 Primer Sets for QRT-PCR and Mice Genotyping**	
**Target gene**	**Oligonucleotides (5'-3')**
homo TNF-a-F	CCTCTCTCTAATCAGCCCTCTG
homo TNF-a-R	GAGGACCTGGGAGTAGATGAG
homo IL-17A-F	CTCATTGGTGTCACTGCTA
homo IL-17A-R	CGGTTATGGATGTTCAGGTT
homo GAPDH-F	TGCACCACCAACTGCTTAGC
homo GAPDH-R	GGCATGGACTGTGGTCATGAG
mus ACTB-F	CCTCTATGCCAACACAGTGC
mus ACTB-R	ACATCTGCTGGAAGGTGGAC
mus TNF-α-F	GCCACCACGCTCTTCTGTCT
mus TNF-α-R	ACTCCAGCTGCTCCTCCACTT
Sigirr-F1	AGTCATCTATGTTTCGGGTTCCG
Sigirr-R1	TCATACTCCCAACCAGGAGGG
Sigirr-F2	AGACTGGCTGTCGATCTTGGAG
