# Supplementary material for: SIGIRR deficiency contributes to CD4 T cell abnormalities by facilitating the IL1/C/EBPβ/TNF-α signaling axis in rheumatoid arthritis
Source: Mol Med. 2022 Nov 18;28:135. doi: 10.1186/s10020-022-00563-9 (PMC9673409; doi:10.1186/s10020-022-00563-9)
Supplement: Supplementary file 13 — Additional file 13: Table S6. Antibodies Used for Immunoblots. [file 10020_2022_563_MOESM13_ESM.pdf]

Supplemental Table 6 Antibodies Used for Immunoblots		
**Antibody**	**Manufacturer**	**Cat. No.**
Phospho-C/EBPβ (Thr235) Antibody	Cell Signaling Technology (CST)	#3084
C/EBPβ (LAP) Antibody	Cell Signaling Technology (CST)	#3087
Phospho-p38 MAPK (Thr180/Tyr182) (D3F9) XP® Rabbit mAb	Cell Signaling Technology (CST)	#4511
p38 MAPK Antibody	Cell Signaling Technology (CST)	#9212
Phospho-SAPK/JNK (Thr183/Tyr185)	Cell Signaling Technology (CST)	#9251
SAPK/JNK Antibody	Cell Signaling Technology (CST)	#9252
Phospho-IκBα (Ser32) (14D4) Rabbit mAb	Cell Signaling Technology (CST)	#2859
IκBα (44D4) Rabbit mAb	Cell Signaling Technology (CST)	#4812
Phospho-p44/42 MAPK (Erk1/2) (Thr202/Tyr204) Antibody	Cell Signaling Technology (CST)	#9101
p44/42 MAPK (Erk1/2) (137F5) Rabbit mAb	Cell Signaling Technology (CST)	#4695
Phospho-NF-κB p65 (Ser536) (93H1) Rabbit mAb	Cell Signaling Technology (CST)	#3033
NF-κB p65 (D14E12) XP® Rabbit mAb	Cell Signaling Technology (CST)	#8242
SIGIRR Polyclonal Antibody	Thermo Fisher SCIENTIFIC	PA5-98507
GFP-tag Antibody	Affinity Biosciences	T0005
GAPDH Antibody	Affinity Biosciences	AF7021
Anti-mouse IgG, HRP-linked Antibody	Cell Signaling Technology (CST)	#7076
Anti-rabbit IgG, HRP-linked Antibody	Cell Signaling Technology (CST)	#7074
